# Supplementary material for: Identification and analysis of splicing quantitative trait loci across multiple tissues in the human genome
Source: Nat Commun. 2021 Feb 1;12:727. doi: 10.1038/s41467-020-20578-2 (PMC7851174; doi:10.1038/s41467-020-20578-2)
Supplement: Supplementary file 1 — Supplementary Information [file 41467_2020_20578_MOESM1_ESM.pdf]

Supplementary Information

Identification and analysis of splicing quantitative trait loci across multiple tissues in the human genome

Diego Garrido-Martín*[1], Beatrice Borsari[1], Miquel Calvo[2], Ferran Reverter[2] and Roderic Guigó*[1,3]

[1]Centre for Genomic Regulation (CRG), The Barcelona Institute of Science and Technology, Dr. Aiguader 88, Barcelona 08003, Catalonia, Spain
[2]Section of Statistics, Faculty of Biology, Universitat de Barcelona (UB), Av. Diagonal 643, Barcelona 08028, Spain
[3]Universitat Pompeu Fabra (UPF), Barcelona, Catalonia, Spain

*Correspondence should be addressed to e-mail: roderic.guigo@crg.eu (Roderic Guigó) or diego.garrido@crg.eu (Diego Garrido-Martín).

Supplementary Figures and Tables

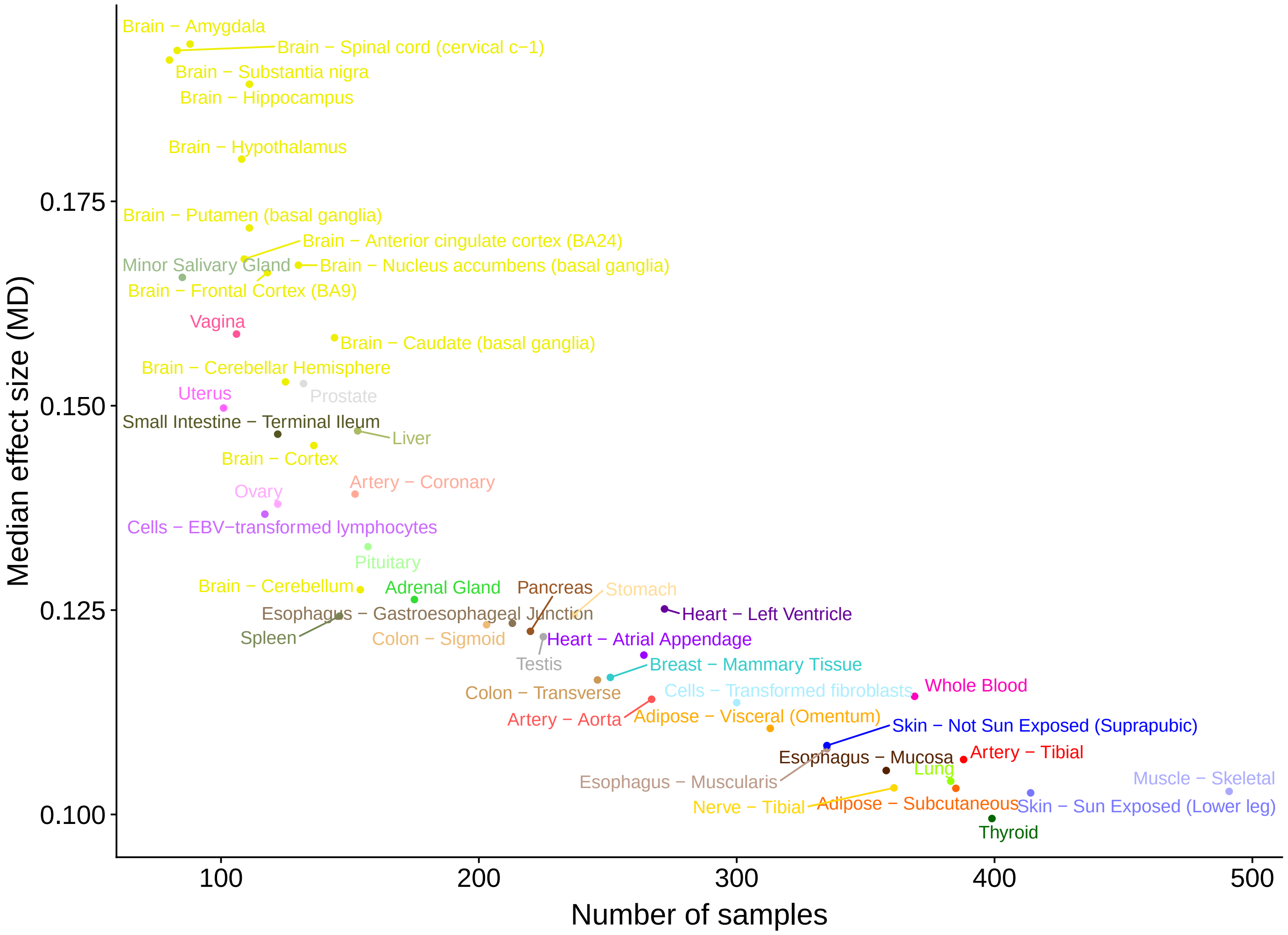

Supplementary Figure 1. sQTL effect size with respect to tissue sample size. Median sQTL effect size (absolute maximum difference in adjusted transcript relative expression between genotype groups, MD), computed for each tissue (y-axis), with respect to the tissue sample size (x-axis). Source data are provided as a Source Data file.

a

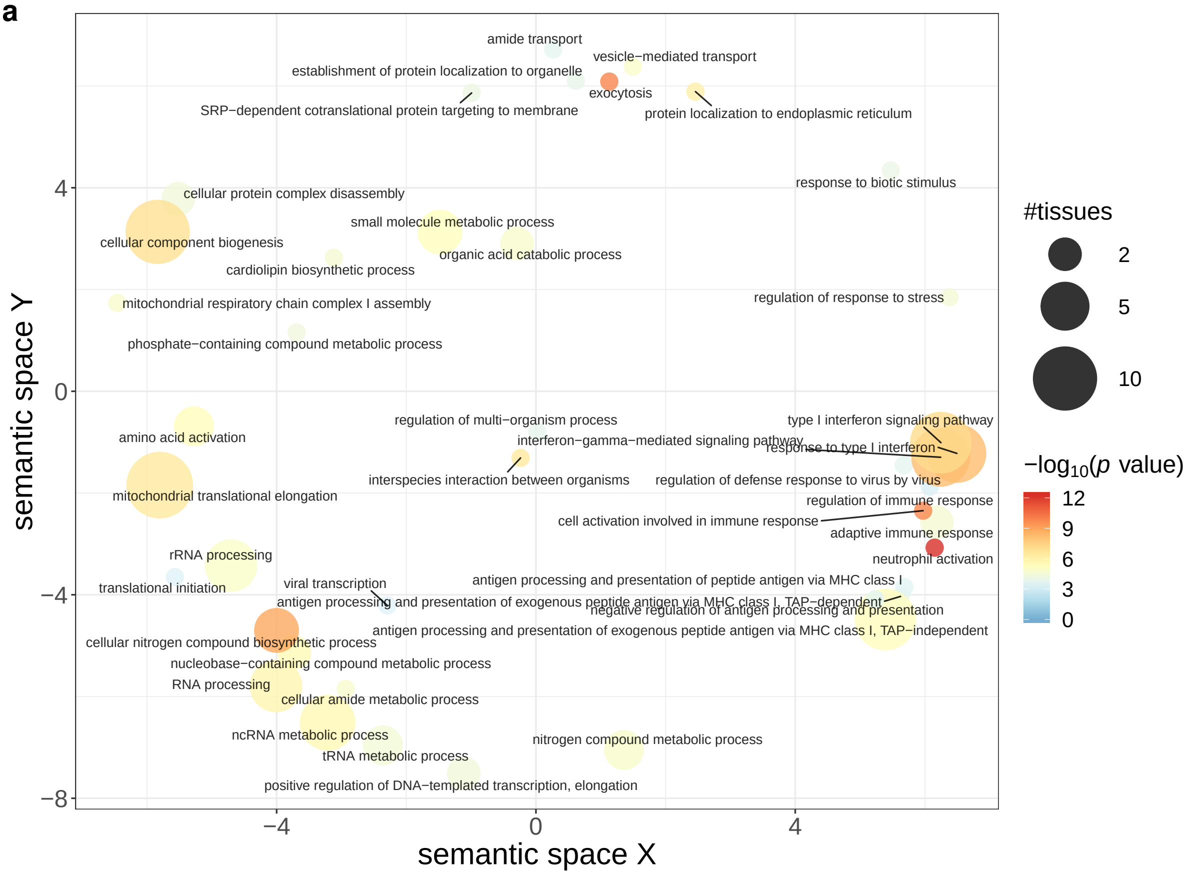

b

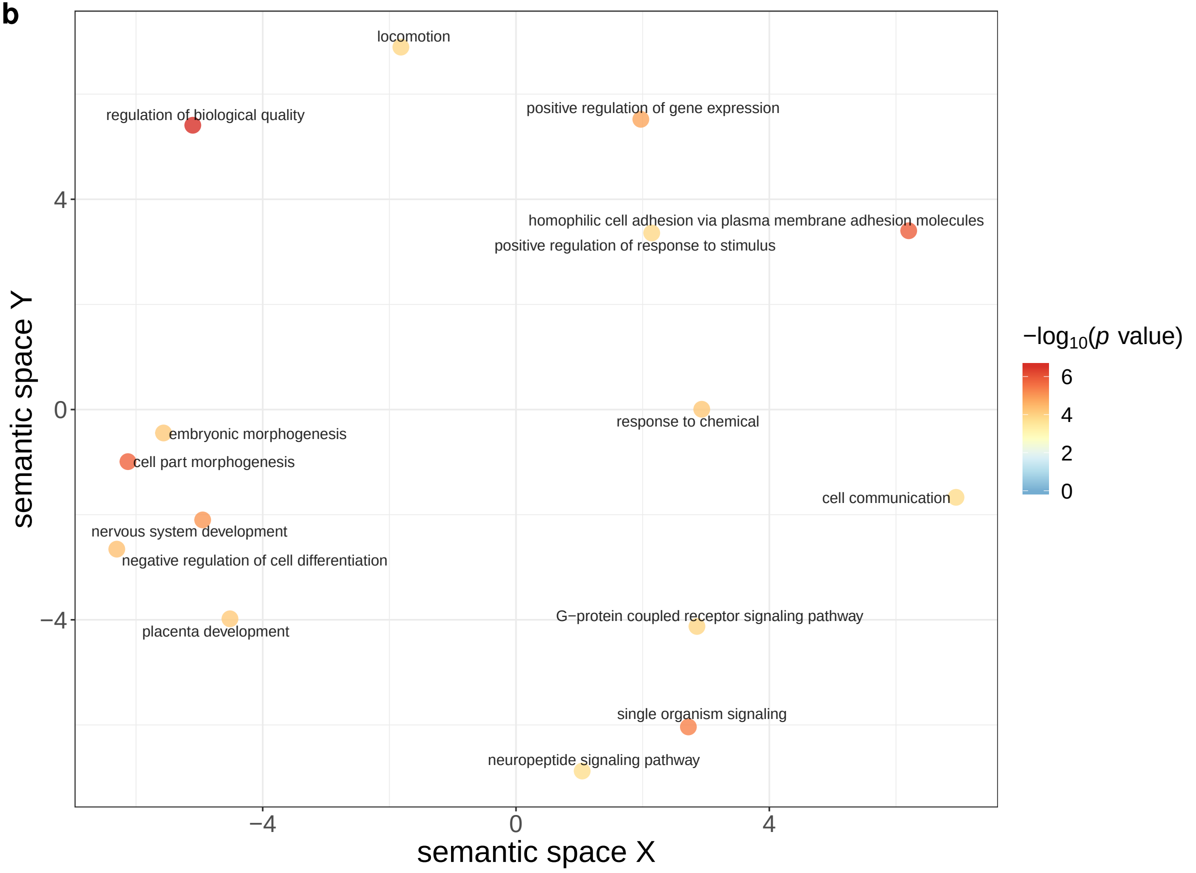

Supplementary Figure 2. Gene Ontology (GO) enrichment analysis. a) Multidimensional scaling-based representation of the semantic dissimilarities between non-redundant GO (Biological Process) terms enriched among sGenes. Each term is represented by a circle, being its size the number of tissues in which the term is enriched and its color the minimum $-\log_{10}$ *p* value (hypergeometric test) for the term enrichment across tissues. GO terms that lie close to each other are semantically more similar. **b)** Analogous representation for genes without sQTLs in any tissue. Source data for both a) and b) are provided as a Source Data file.

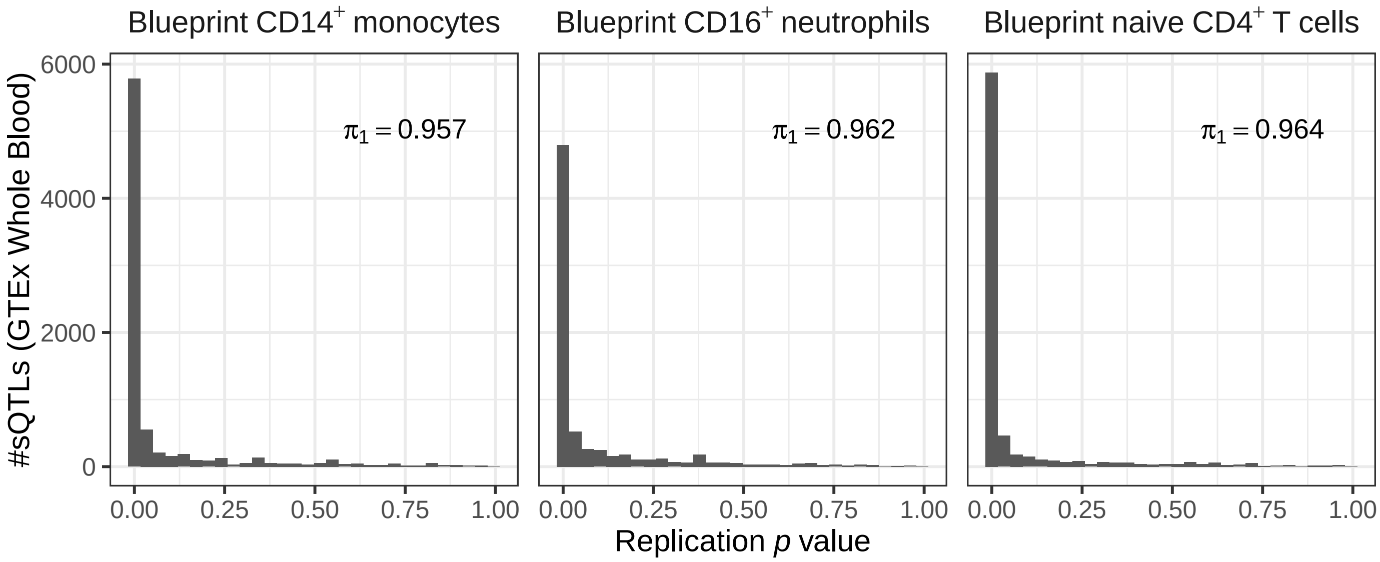

Supplementary Figure 3. sQTL replication. Histogram of the replication *p* values (Anderson test) of GTEx whole blood sQTLs in three immune cell types ($CD14^+$ monocytes, $CD16^+$ neutrophils, and naive $CD4^+$ T cells) from the Blueprint Project. π_1 statistics are also shown. Source data are provided as a Source Data file.

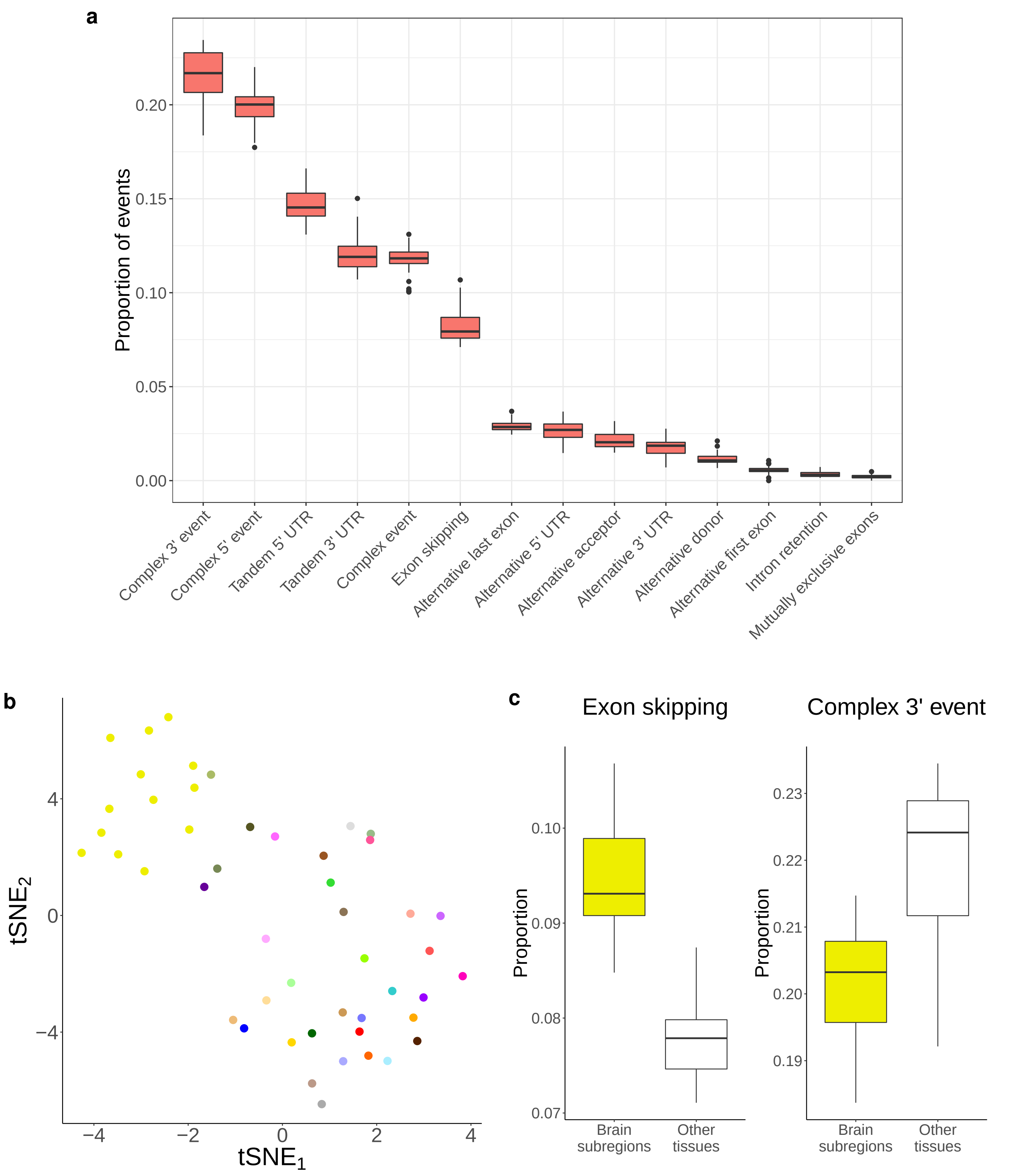

Supplementary Figure 4. Alternative splicing (AS) events associated with sQTLs. a) Distribution of the proportion of AS events of each type associated with sQTLs across 48 GTEx tissues (see Methods). **b)** Representation in two dimensions, for each tissue, of the vector of proportions of the different types of AS events, obtained by t-SNE. Tissues with a similar pattern of AS events associated with sQTLs are closer in the bi-dimensional space, with brain subregions forming a distinct cluster. **c)** Comparison of the distribution of the proportions of exon skipping and complex 3' events associated with sQTLs in brain (yellow, $n = 13$) and non-brain tissues (white, $n = 35$). Brain subregions display a larger proportion of simple events affecting internal exons and introns, such as exon inclusion (two-sided Wilcoxon Rank-Sum test *p* value $6.94 \cdot 10^{-10}$), and a smaller proportion of events affecting first/last exons and UTRs, such as complex 3' events (two-sided Wilcoxon Rank-Sum test *p* value $3.30 \cdot 10^{-6}$). In a) and c), data is shown as boxplots, where the box represents the first to third quartiles and the median, and the whiskers indicate $\pm$ 1.5 $\times$ interquartile range (IQR). Source data for a) – c) are provided as a Source Data file.

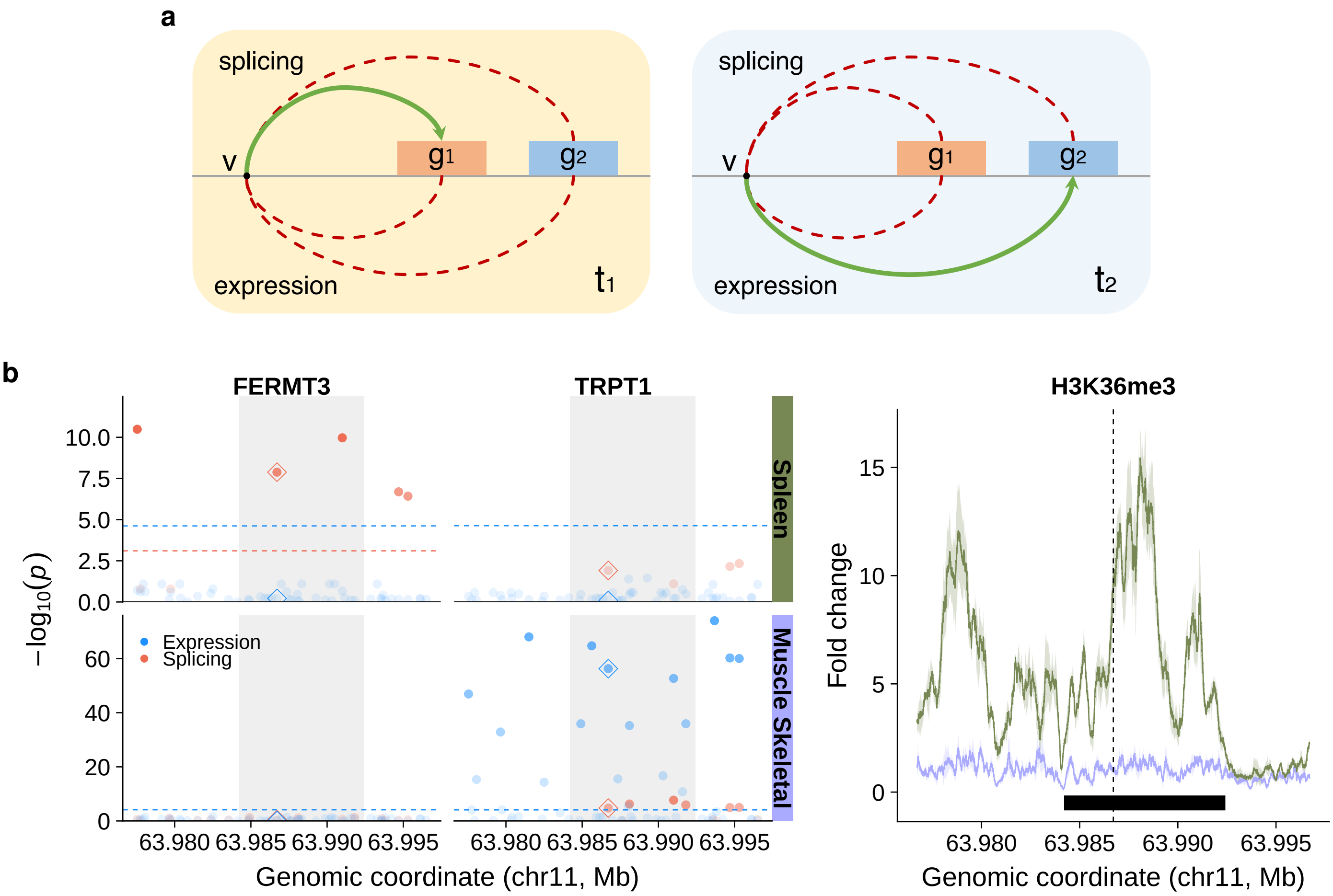

Supplementary Figure 5. Heteropleiotropy. a) A variant v is considered heteropleiotropic in two different tissues (i.e. t_1 and t_2) if: i) it is an sQTL, but not an eQTL, for gene g_1 in tissue t_1, ii) it is an eQTL, but not an sQTL, for gene g_2 in tissue t_2, iii) it is neither an sQTL nor an eQTL for gene g_2 in tissue t_1, iv) it is neither an sQTL nor an eQTL for gene g_1 in tissue t_2. **b)** Example of a heteropleiotropic locus. The SNP rs11603538 (chr11:63,986,713, C/A) is an sQTL for the gene *FERMT3* (chr11:63,974,150-63,991,354, forward strand) in Spleen ($n = 146$), but not in Muscle Skeletal ($n = 491$). The SNP is not an eQTL for *FERMT3* in any of the two tissues. In contrast, the SNP is an eQTL for the gene *TRPT1* (chr11:63,991,272-63,993,726, reverse strand) in Muscle Skeletal, but not in Spleen. The SNP is not an sQTL for *TRPT1* in any of the two tissues. In the left panel, the dots represent the $-\log_{10}$ *p* values of association with the expression (two-sided *t*-test, blue) and splicing (Anderson test, red) of the two genes in the two tissues, for variants in a 20 Kb window centered at rs11603538 (the $-\log_{10}$ *p* values corresponding to rs11603538 are highlighted by a diamond). The transparency of the dots depends on the $-\log_{10}$ *p* value. The significance level for each molecular trait, gene and tissue is shown as a coloured, horizontal dashed line. When this line is not present, the gene-level *p* value is above the 0.05 FDR threshold and hence no variant is significantly associated with this molecular trait in this tissue (see Methods). The shaded area represents the position of a H3K36me3 ChIP-seq peak (see below). The right panel shows the fold-change signal of the H3K36me3 histone mark with respect to the input across three ENTEx donors in Spleen and Muscle Skeletal, in the same genomic region of the left panel. The line and the coloured area correspond, respectively, to the mean fold-change signal and its standard error (SEM) across three ENTEx donors (i.e. mean $\pm$ SEM). The location of the SNP (vertical dashed line) and the overlapping ChIP-seq peak (intersection of the peaks in the three donors, black rectangle) are also displayed. Source data are provided as a Source Data file.

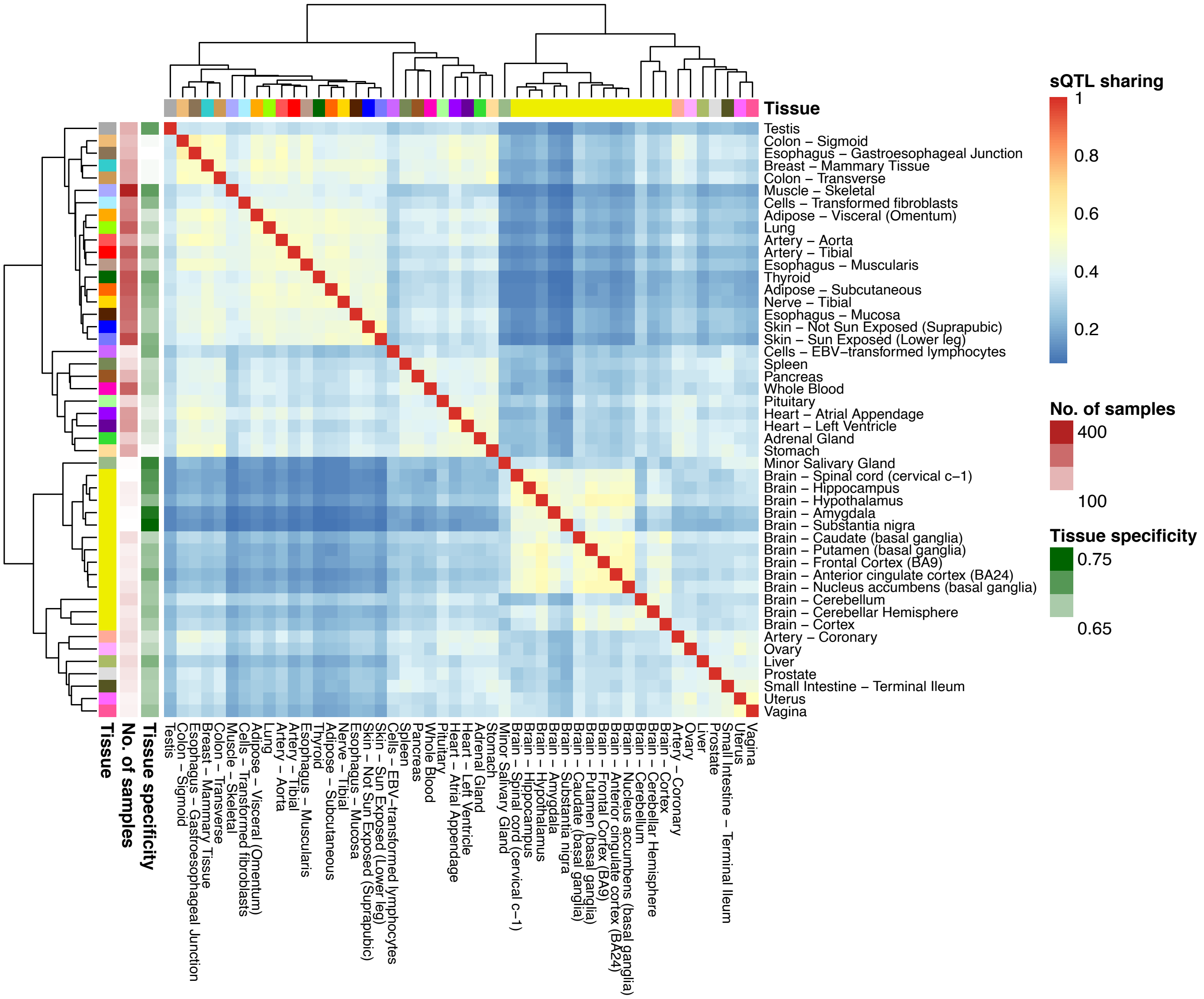

Supplementary Figure 6. sQTL sharing measured by Jaccard index. Heatmap showing sQTL sharing patterns across GTEx tissues. Jaccard index computed on exact variant-gene pairs is employed as sharing estimate. Tissue specificity is estimated as $1 - \bar{j}_t$, where $\bar{j}_t$ is the mean Jaccard index between a given tissue t and the others. Hierarchical clustering of the tissues based on the sharing patterns is also displayed, together with the corresponding tissue colors, sample sizes and tissue specificity estimates. Source data are provided as a Source Data file.

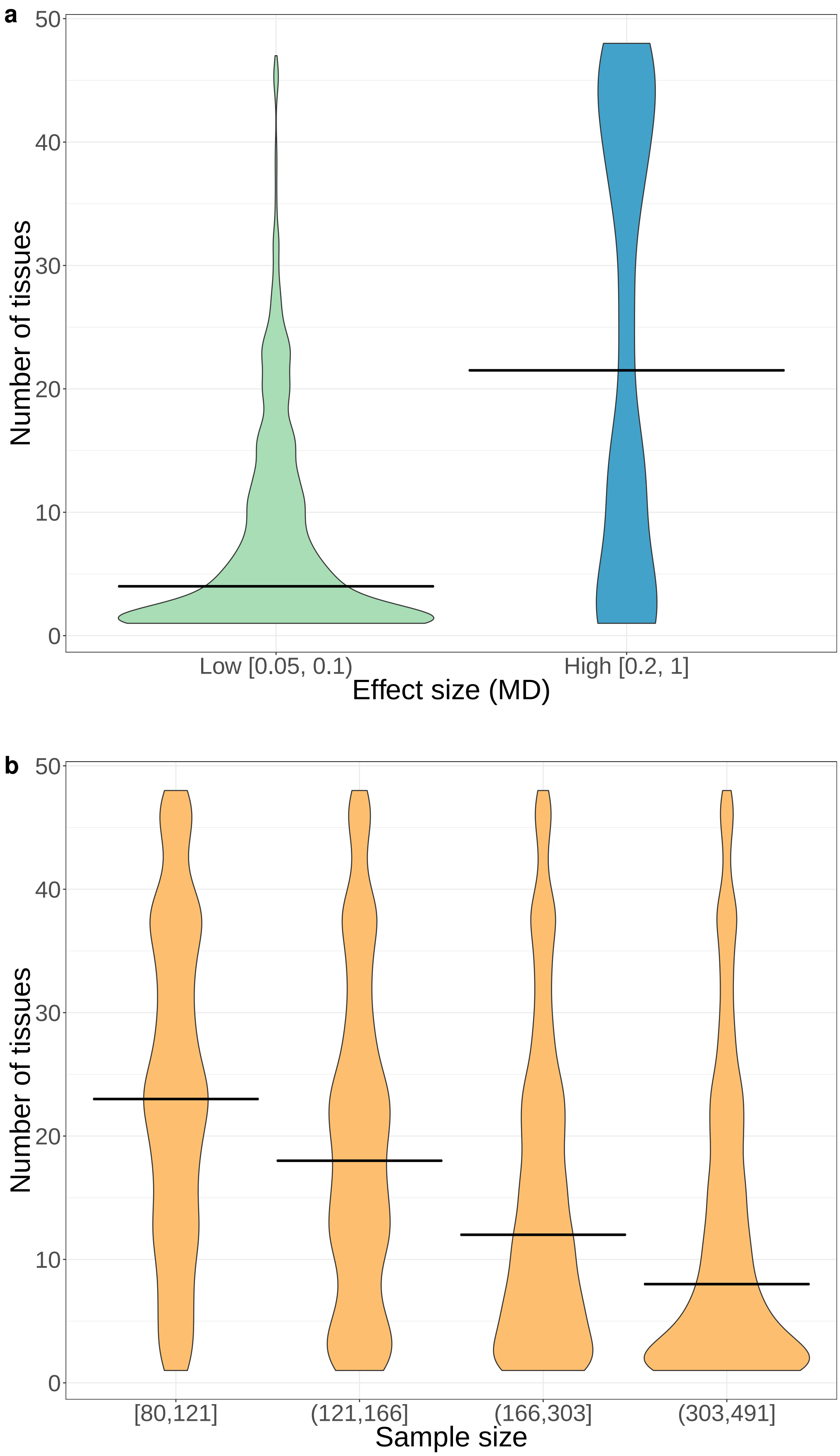

Supplementary Figure 7. sQTL sharing with respect to sQTL effect size and tissue sample size. For all variant-gene pairs tested in all GTEx tissues with $n \geq 70$, we show the distribution of the number of tissues in which the variants are identified as sQTLs for the target genes separately **a)** for high (MD $\geq$ 0.2) and low (MD $<$ 0.1) effect size sQTLs and **b)** for each quartile of the tissue sample size distribution (note that the same variant-gene pair can be included in more than one quartile group). In both a) and b), the median number of tissues is displayed as a black horizontal line. Source data for both a) and b) are provided as a Source Data file.

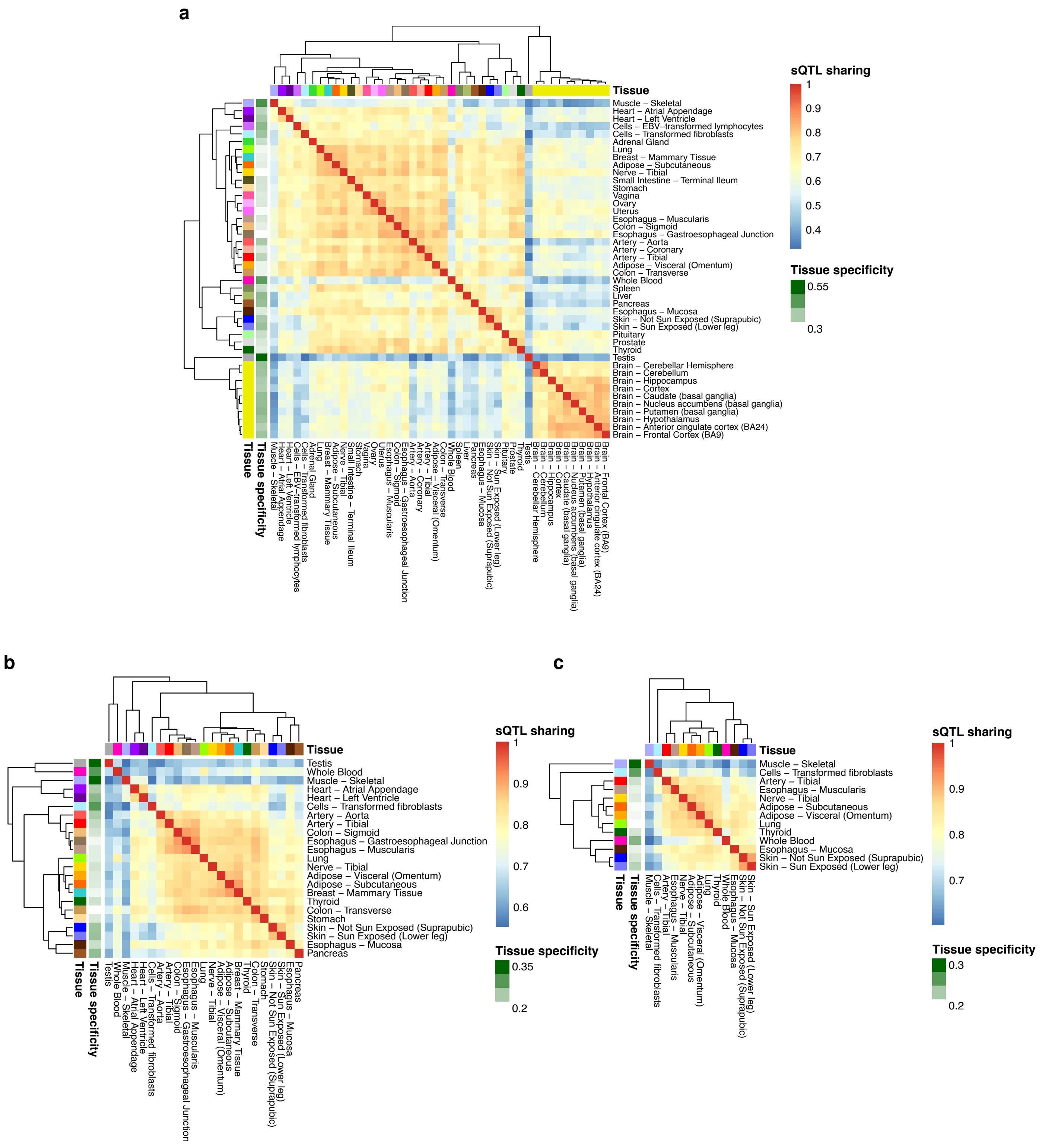

Supplementary Figure 8. sQTL sharing in downsampled GTEx datasets. GTEx tissues were randomly downsampled once to **a)** 100 **b)** 200 and **c)** 300 samples, respectively (see Methods). Sharing estimates range from 0 (low sharing, blue) to 1 (high sharing, red). In addition, we display the hierarchical clustering of the tissues based on sQTL sharing, together with the tissue specificity estimates. Source data for a) – c) are provided as a Source Data file.

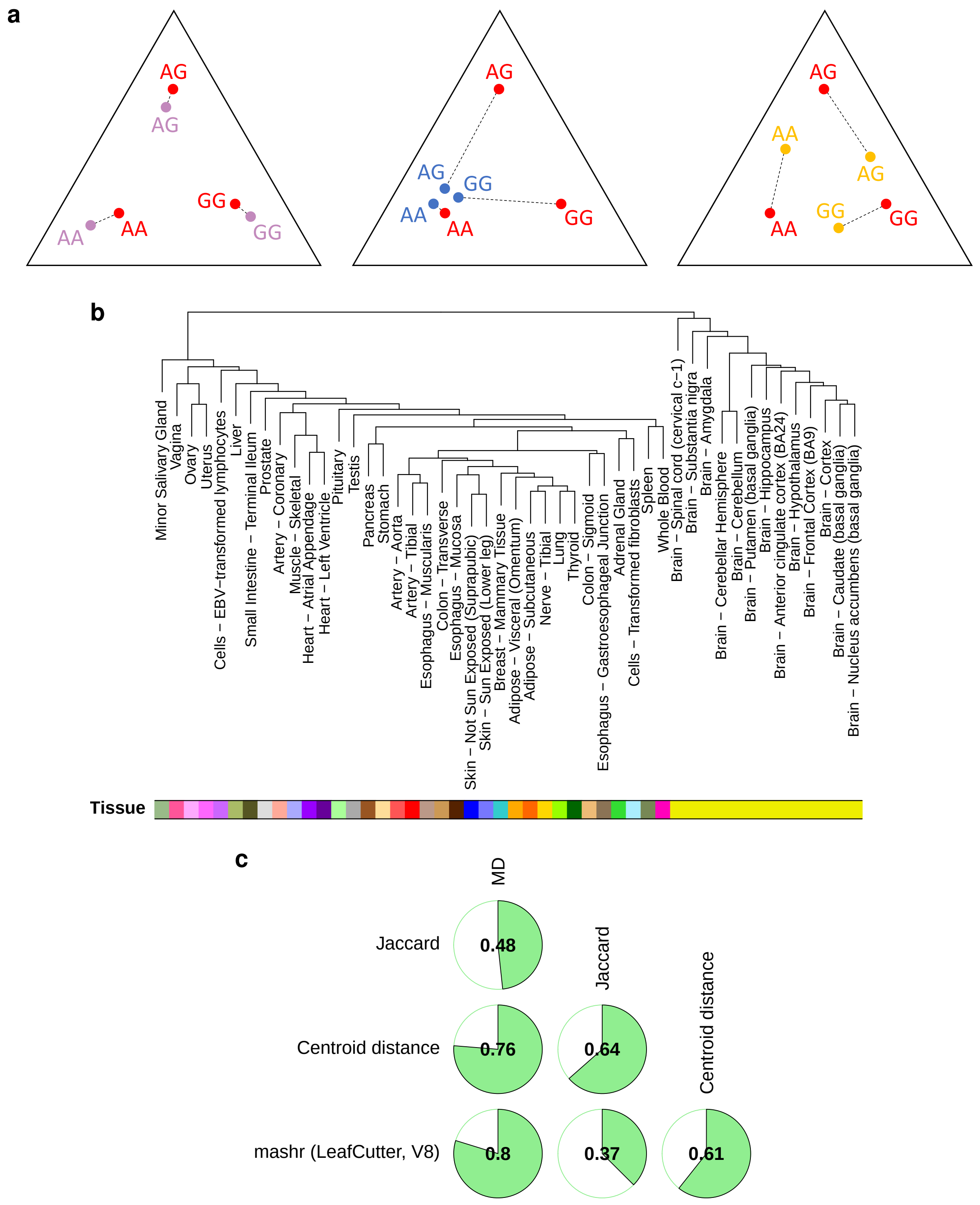

Supplementary Figure 9. Centroid distance approach and comparison between strategies to assess sQTL sharing. a) Illustrative example of the centroid distance approach for sQTL sharing (see Methods). A SNP with alleles A>G is an sQTL for a gene with three isoforms in a given tissue t_1 (red). The average individual (centroid) of each genotype group can be represented as a point in a three-dimensional space, whose coordinates are the mean relative abundances of each isoform across individuals in this genotype group. As relative abundances add up to one, these points are located on a two-dimensional simplex (i.e. a triangle). The left panel shows a SNP that is an sQTL for the same gene in tissue t_2 (violet), affecting isoform abundances in the same way as in tissue t_1 (red). Note that here we consider a single variant-gene pair ($p = 1$), and d is given by the sum of the lengths of the dashed lines. Hence, in this case $d(t_1, t_2)$ would be small. In the middle panel, the SNP is not an sQTL for the same gene in tissue t_3 (blue), and therefore $d(t_1, t_3)$ would be large. In the right panel, the SNP is an sQTL for the same gene in tissue t_4 (yellow), but the associated change in splicing isoform abundances is different, and $d(t_1, t_4)$ would be large. **b)** Hierarchical clustering built using the centroid distance approach. Source data are provided as a Source Data file. **c)** Estimates of similarity (Baker's Gamma) between tissue dendrograms obtained using MD correlations, the Jaccard index and the centroid distance approach. The dendrogram obtained using mashr on LeafCutter sQTLs from GTEx V8 is also compared. All similarity estimates are significantly different from 0 (permutation test *p* value $< 10^{-4}$).

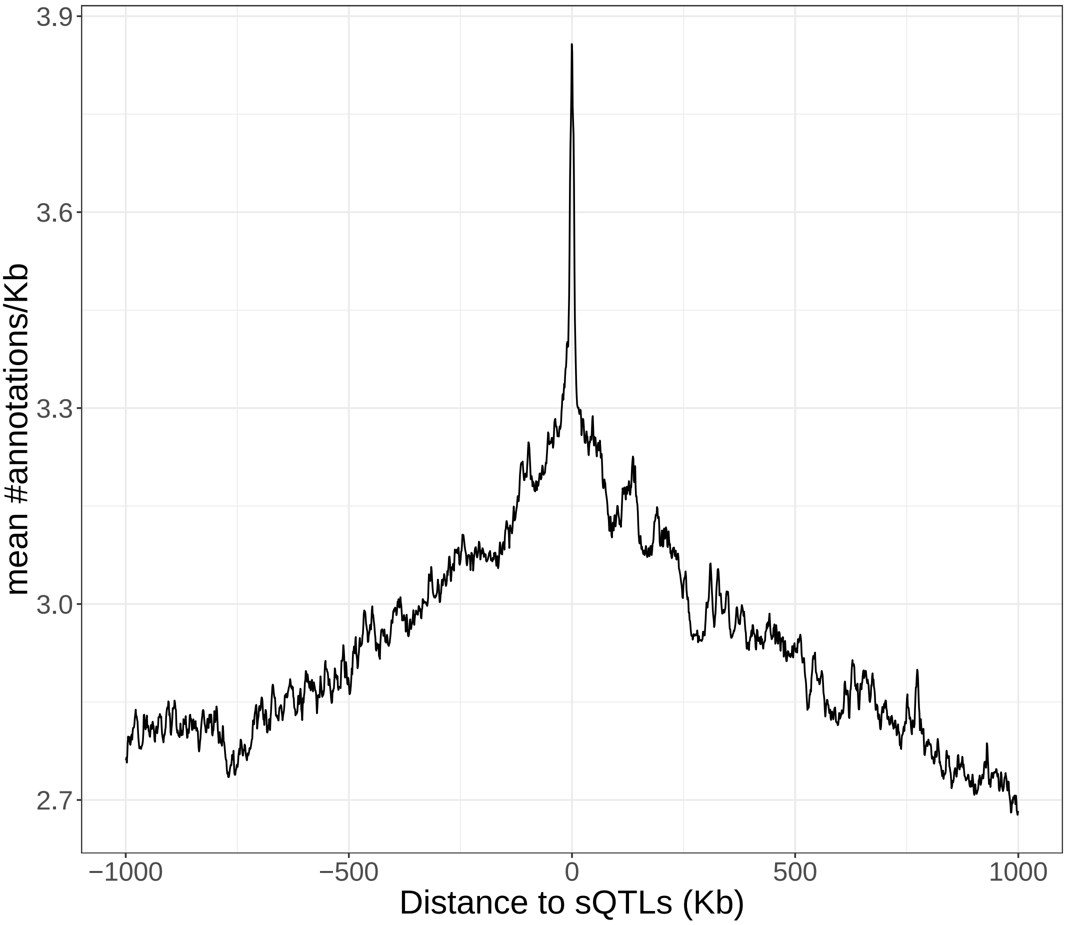

Supplementary Figure 10. Density of functional annotations around sQTLs. Density of functional annotations (mean number of annotations per Kb, y-axis) with respect to the distance to sQTLs (x-axis). The location of the different functional elements was obtained from the Ensembl Regulation dataset (see Methods). The distance 0 corresponds to the functional annotations that overlap sQTLs. Source data are provided as a Source Data file.

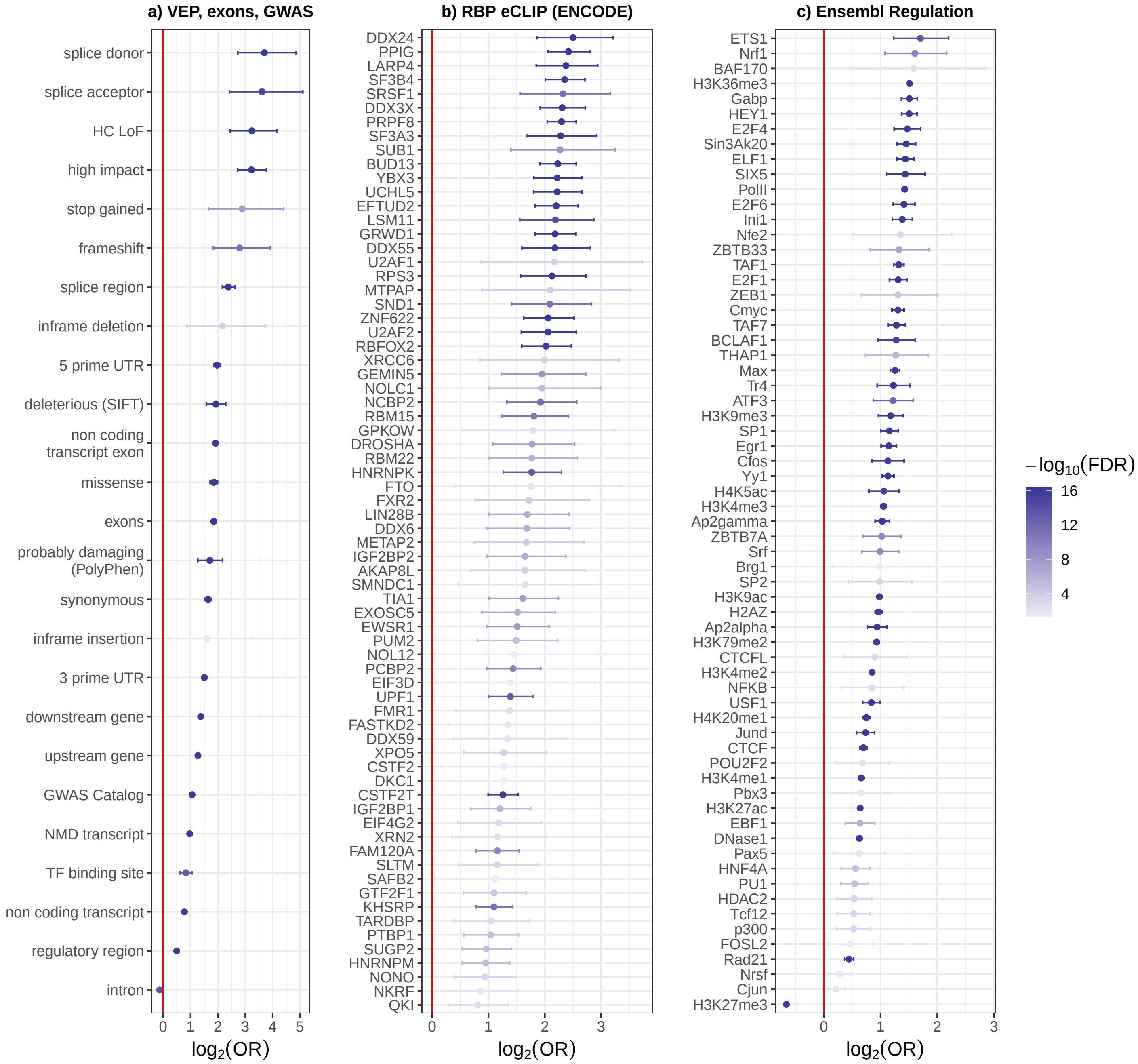

Supplementary Figure 11. Enrichment of sQTLs in functional annotations. Significant enrichment of sQTLs with respect to non-sQTLs (two-sided Fisher's exact test with n = 211,173, FDR $<$ 0.05) in several functional annotations: **a)** Variant Effect Predictor categories and impact, GENCODE v19 protein coding and lincRNA exons and GWAS Catalog; **b)** ENCODE RBP eCLIP peaks and **c)** Ensembl Regulation data (see Methods). For each functional element, the dot represents the enrichment $\log_2$ odds ratio (OR), and the error bar its 95% confidence interval. FDR values are color-coded. Source data for a) – c) are provided as a Source Data file.

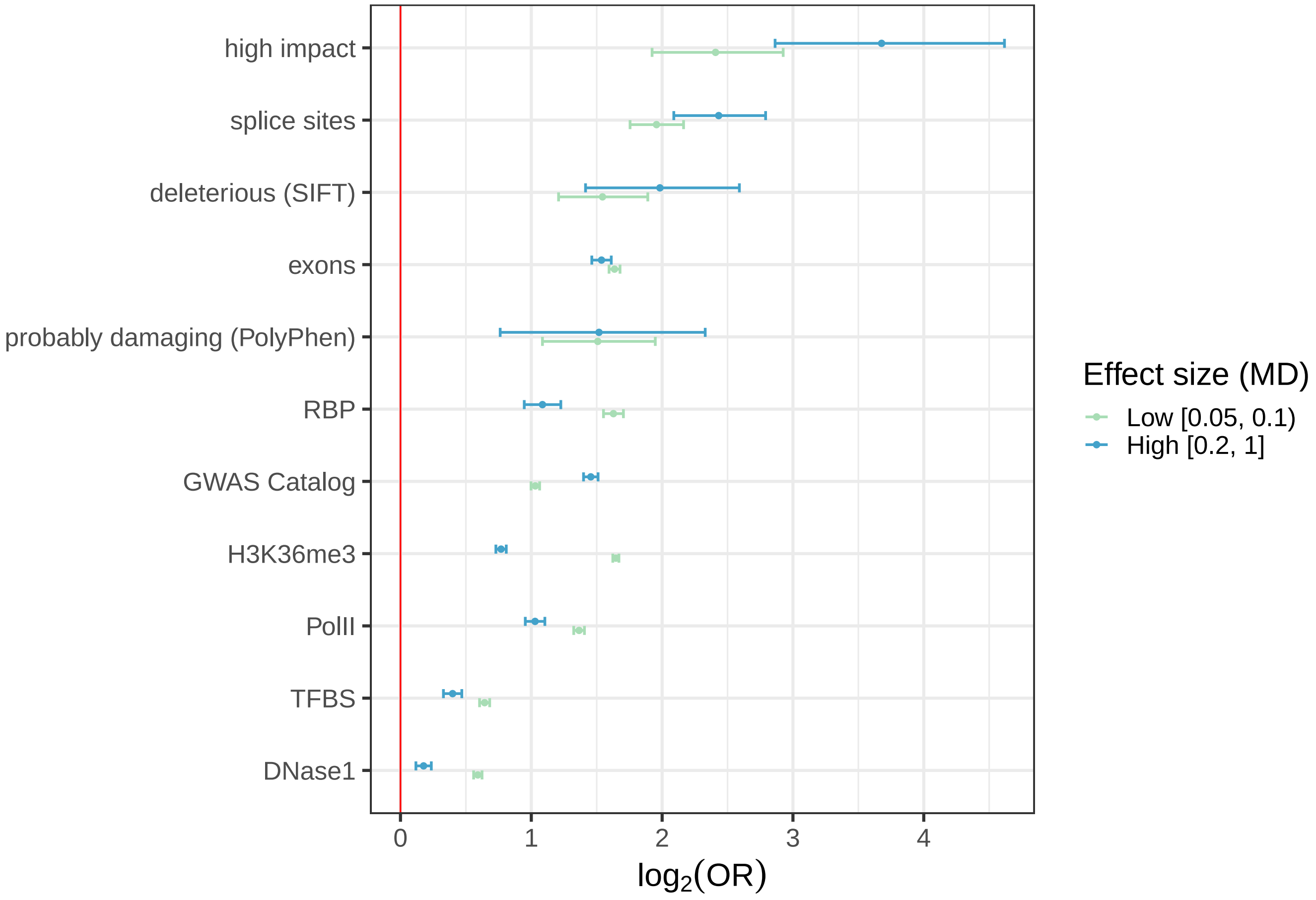

Supplementary Figure 12. Enrichment of high and low effect size sQTLs in functional annotations. Comparison of the enrichment of high (MD $\geq$ 0.2) and low (MD $<$ 0.1) effect size sQTLs (with respect to non-sQTLs, two-sided Fisher's exact test FDR $<$ 0.05, $n_{\text{high}} = 84{,}449$ and $n_{\text{low}} = 256{,}288$) in a set of functional categories. For each effect size group and functional category, the dot represents the enrichment $\log_2$ odds ratio (OR), and the error bar its 95% confidence interval. TFBS and RBP correspond to pooled transcription factor and RBP binding sites, respectively. Source data are provided as a Source Data file.

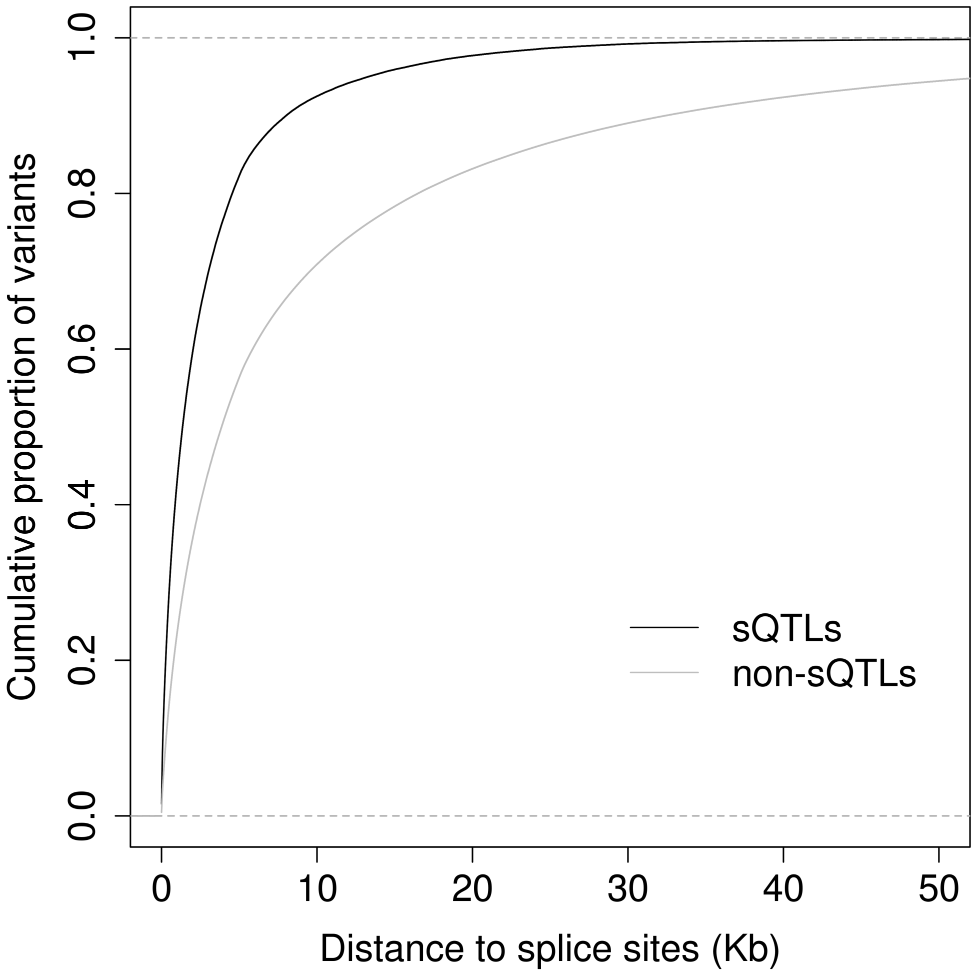

Supplementary Figure 13. Distance of sQTLs to splice sites. Cumulative distribution of the distance to the closest splice donor or acceptor site from protein coding and lincRNA genes annotated in GENCODE v19, both for sQTLs and non-sQTLs. Source data are provided as a Source Data file.

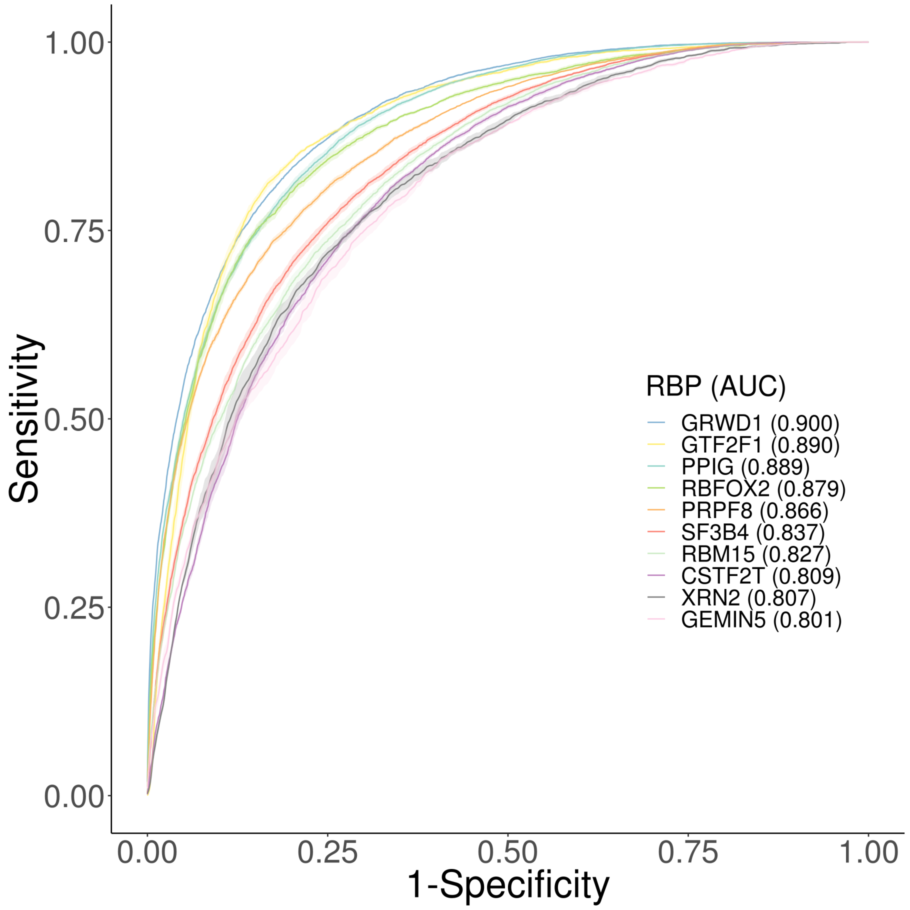

Supplementary Figure 14. gkm-SVM classification performance. Receiver Operating Characteristic (ROC) curves for ten RBPs corresponding to the classification of their eCLIP peaks by a gapped k-mer support vector machine (gkm-SVM). For each RBP, the line and the coloured area correspond, respectively, to the mean and its standard error across the cross-validation folds. The mean area under the curve (AUC) for each RBP is shown between parentheses. Source data are provided as a Source Data file.

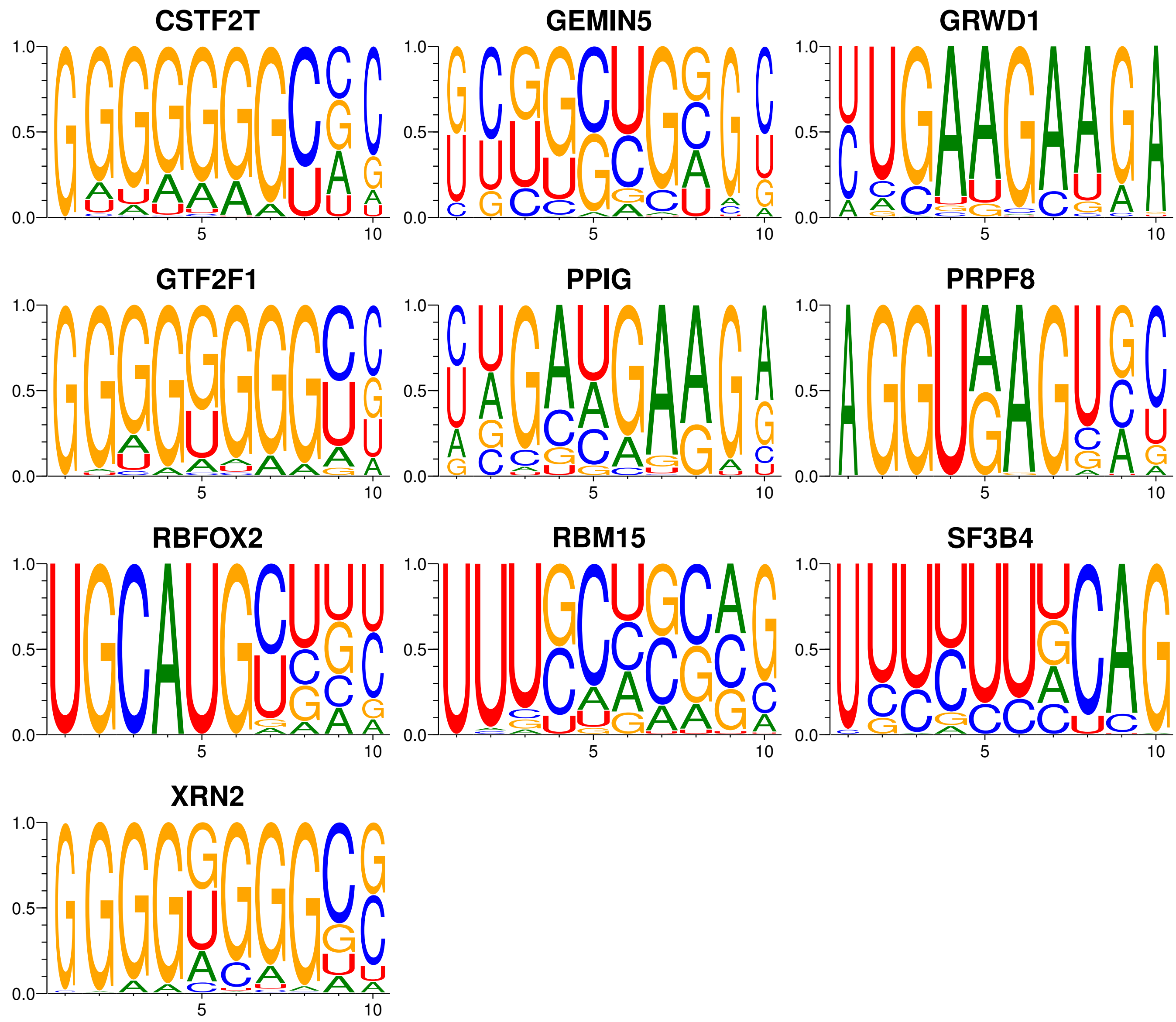

Supplementary Figure 15. RBP binding motif logos. Sequence logos for the predicted binding motifs of ten RBPs, derived from the alignment of the 100 highest-scoring gkm-SVM 10-mers for each RBP. The proportion of each nucleotide (y-axis) is shown for each position of the sequence (x-axis). Source data are provided as a Source Data file.

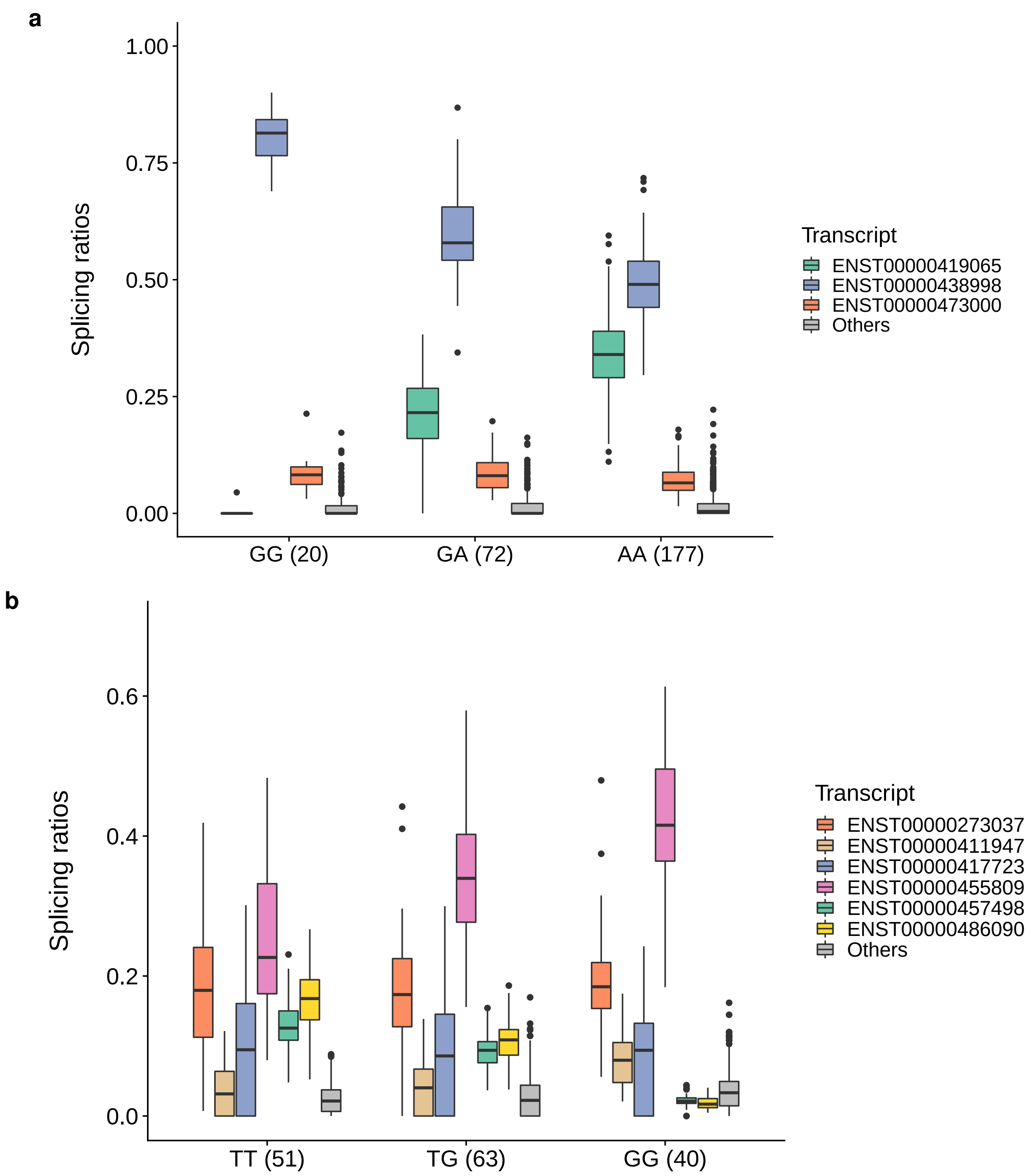

Supplementary Figure 16. Effect of rs4959783 and rs9876026 on the relative transcript abundances of their target genes. a) Relative abundances of the most expressed isoforms in heart (left ventricle) from the gene *PSMG4* (chr6:3,231,637-3,303,607, forward strand), for each genotype group at the rs4959783 locus (chr6:3,260,093, G/A in the forward strand). The least abundant isoforms are grouped in Others. The number of individuals in each genotype group is shown between parentheses (total $n = 269$). In reference homozygous individuals at rs4959783 (GG), isoform ENST00000438998 (blue) captures most of the expression of the gene and isoform ENST00000419065 (green) is not expressed. In contrast, in alternative homozygous individuals (AA) both isoforms have comparable splicing ratios. Heterozygous individuals (GA) display an intermediate behaviour. rs4959783 is an sQTL for *PSMG4* in a total of 8 tissues ($\overline{\text{MD}} = 0.30$). **b)** Analogous representation for the gene *TAMM41* (chr3:11,831,916-11,888,393, reverse strand) and the SNP rs9876026 (chr3:11,849,807, T/G in the reverse strand) in cerebellum ($n = 154$). In this case, the abundance of isoform ENST00000455809 (pink) increases with the number of copies of the alternative allele (G) at rs9876026. In contrast, isoforms ENST00000457498 (green) and ENST00000486090 (yellow) display the opposite behaviour, being less abundant in alternative homozygous individuals. rs9876026 is an sQTL for *TAMM41* in a total of 46 tissues ($\overline{\text{MD}} = 0.18$). Both in a) and b), data is shown as boxplots, where the box represents the first to third quartiles and the median, and the whiskers indicate $\pm$ 1.5 $\times$ interquartile range (IQR). Source data for both a) and b) are provided as a Source Data file.

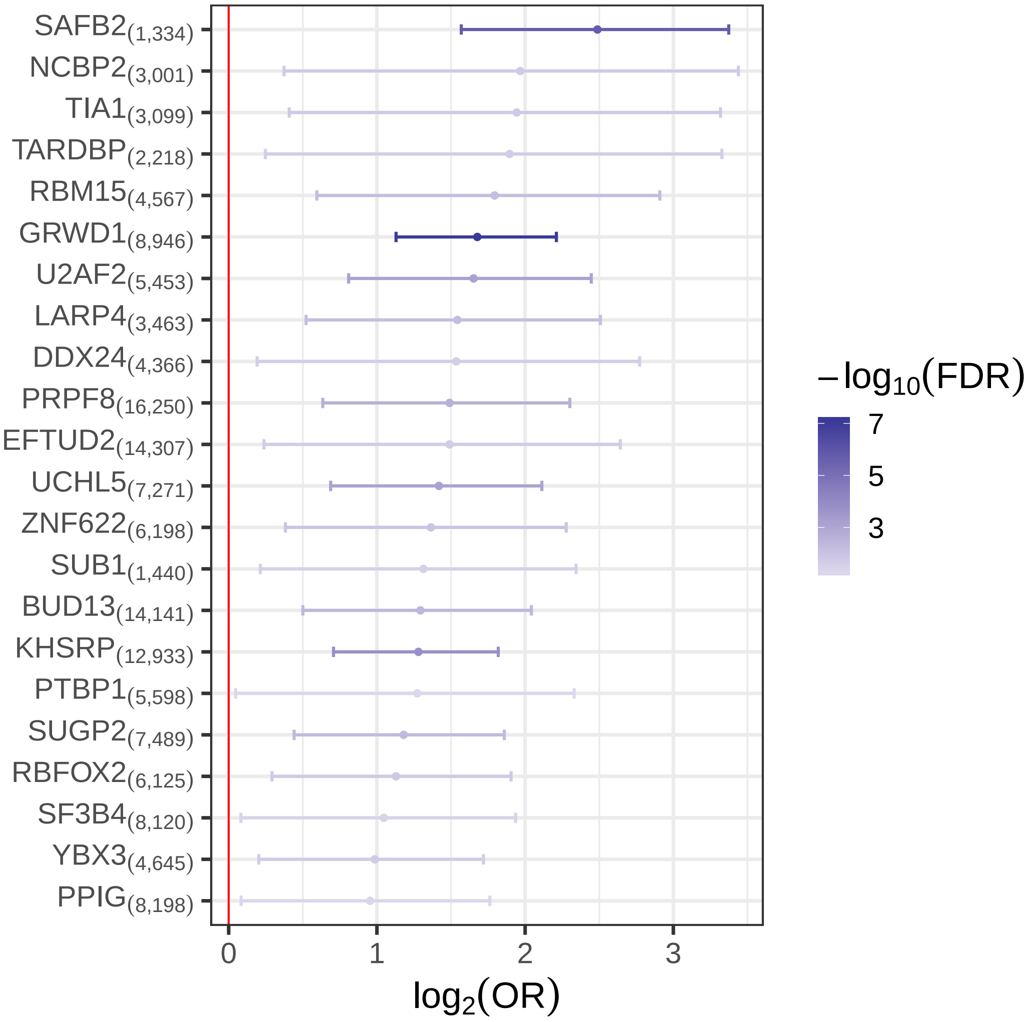

Supplementary Figure 17. Enrichment of sQTLs in allele-specific RBP binding (ASB) variants. Enrichment of sQTLs *vs* matched non-sQTLs in a set of ASB variants identified by BEAPR (Binding Estimation of Allele-specific Protein-RNA interaction) in the ENCODE eCLIP dataset (two-sided Fisher's exact test, FDR < 0.05). For each RBP, the dot represents the enrichment $\log_2$ odds ratio (OR), and the error bar its 95% confidence interval. The total sample size (n) for each test is shown between parentheses. FDR values are color-coded. Source data are provided as a Source Data file.

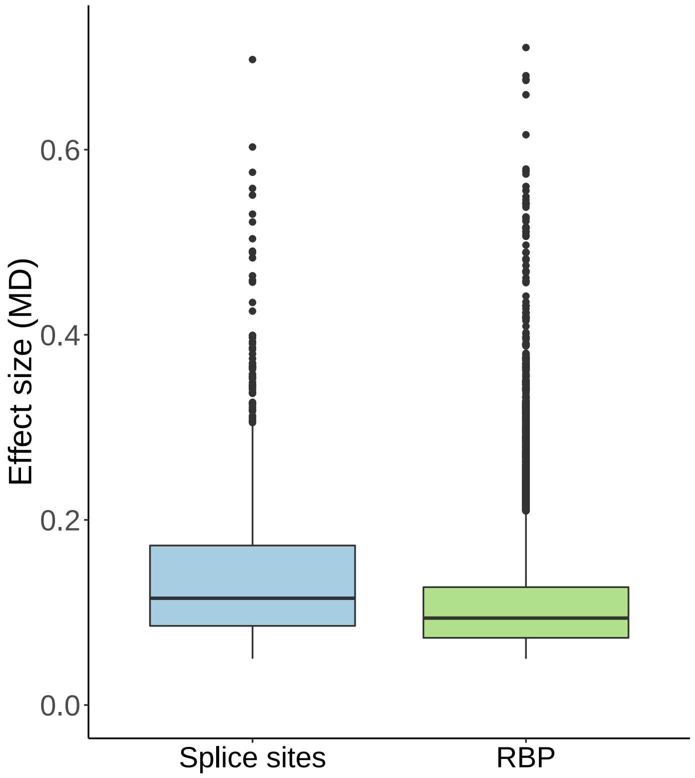

Supplementary Figure 18. **Effect size of sQTLs in splice sites and RBP binding sites.** Comparison of the distribution of effect sizes (MD values) for sQTLs in splice sites and RBP binding sites (pooled RBP eCLIP peaks from ENCODE). Variants falling in both categories have been removed. Data is shown as boxplots, where the box represents the first to third quartiles and the median, and the whiskers indicate $\pm$ 1.5 $\times$ interquartile range (IQR). Two-sided Wilcoxon Rank-Sum test *p* value $< 10^{-16}$ ($n =$ 17,641 sQTLs). Source data are provided as a Source Data file.

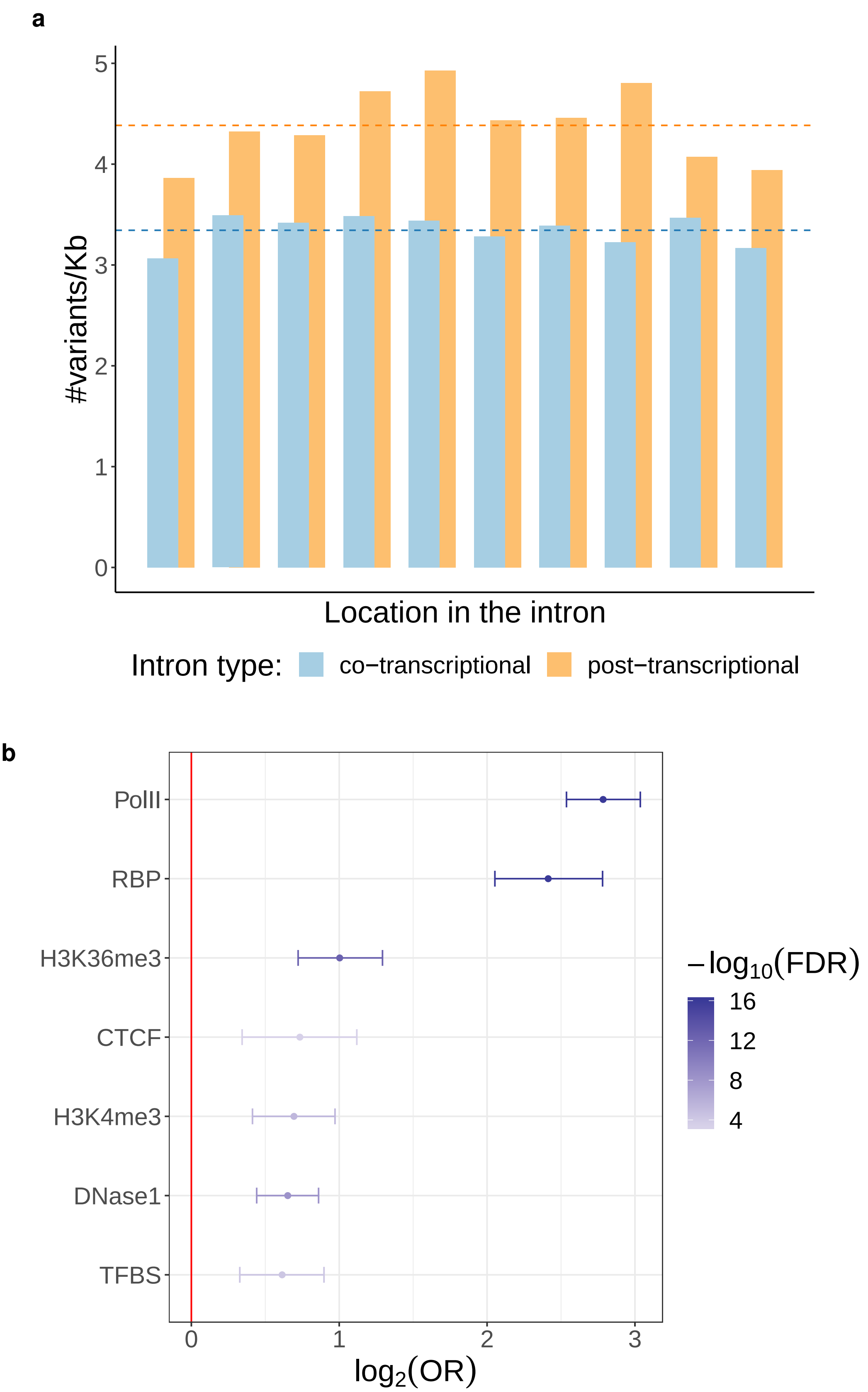

Supplementary Figure 19. sQTLs and co-/post-transcriptional splicing. a) Variant density (number of variants per Kb) along co- (blue) and post-transcriptionally spliced (orange) introns. Horizontal dashed lines represent the average variant density for each group of introns. **b)** Functional enrichment of sQTLs falling in post-transcriptionally spliced introns *vs* sQTLs falling in co-transcriptionally spliced introns (two-sided Fisher's exact test with $n =$ 9,373, FDR $<$ 0.05, odds ratio $\geq$ 1.5). For each functional element, the dot represents the enrichment $\log_2$ odds ratio (OR), and the error bar its 95% confidence interval. FDR values are color-coded. TFBS and RBP correspond to pooled transcription factor and RBP binding sites, respectively. Source data for both a) and b) are provided as a Source Data file.

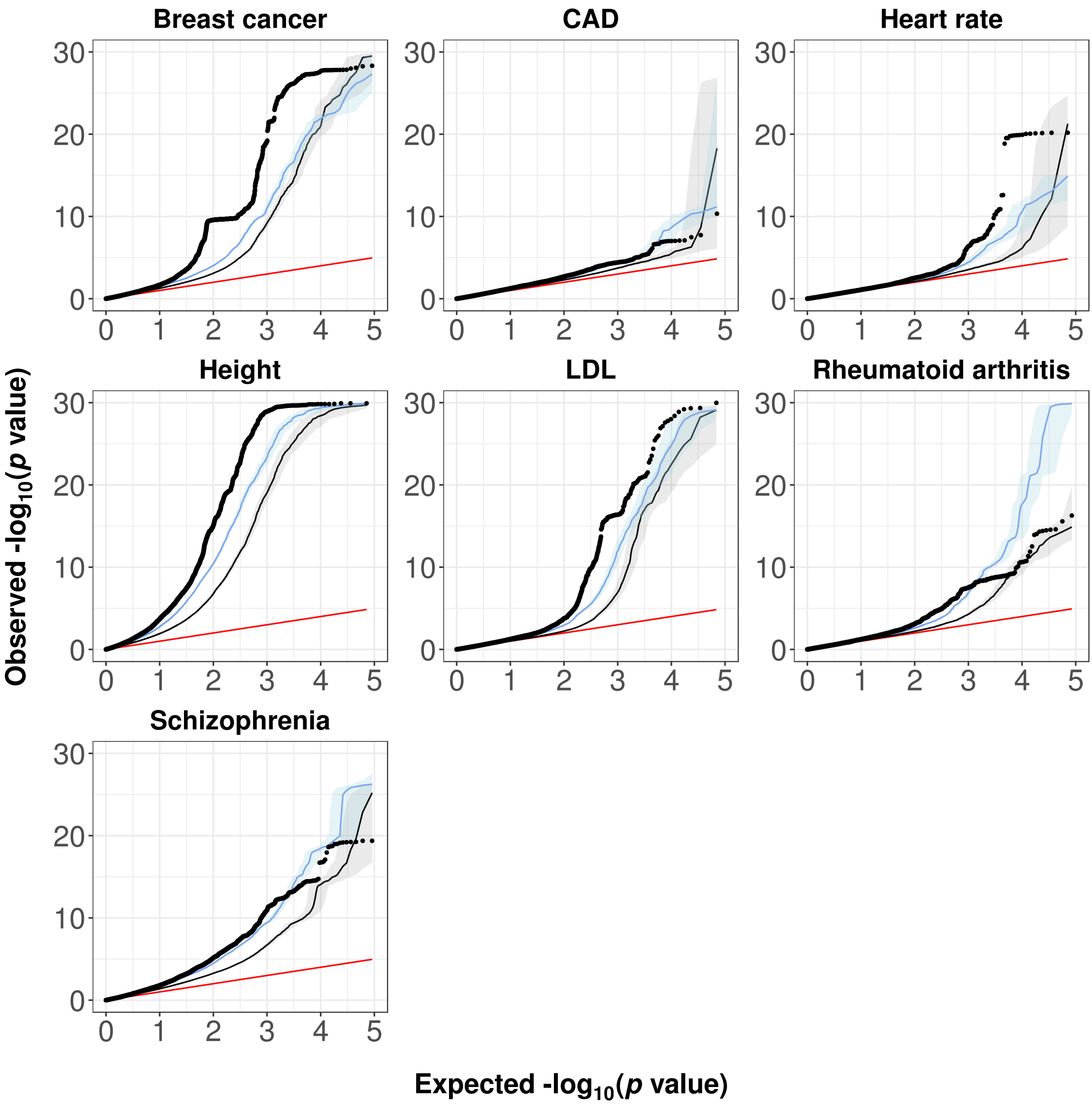

Supplementary Figure 20. sQTLs and GWAS. Quantile-quantile (QQ) plots of *p* values for association with several traits and diseases, including breast cancer, coronary artery disease (CAD), heart rate, height, low-density lipoprotein (LDL) levels, rheumatoid arthritis and schizophrenia, for sQTLs (black dots), eQTLs without effects on splicing (blue line and area), and variants with effects neither on expression nor on splicing (black line and grey area). Lines and coloured areas represent, respectively, medians and middle 95% observed $-\log_{10}$ *p* values across 10,000 random samplings from the corresponding variant set, with the same size as the sQTL set. The identity line is shown in red. Further information on *p* value calculation and other details of each GWAS are available at the corresponding publications, referenced in Methods. Source data are provided as a Source Data file.

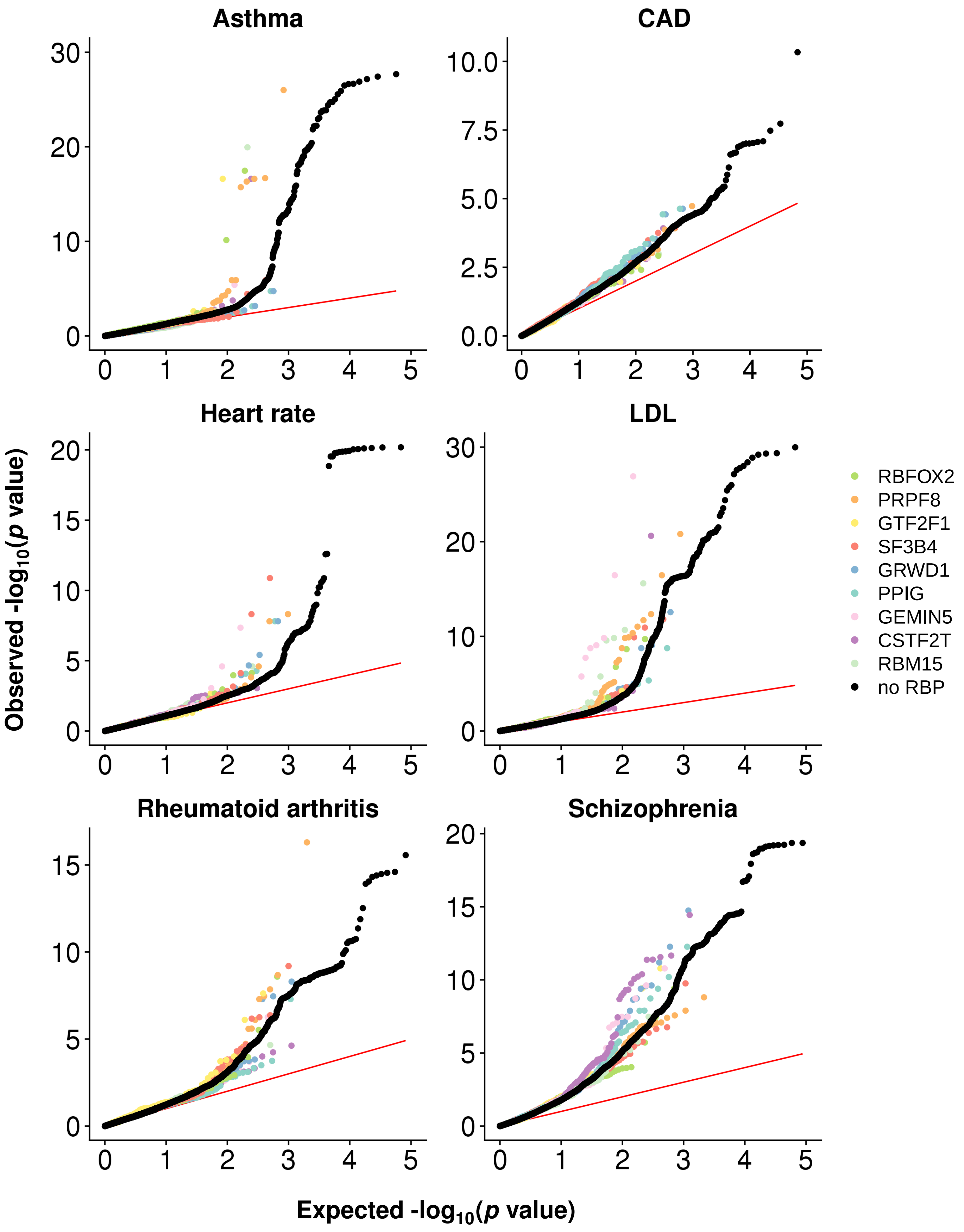

Supplementary Figure 21. sQTLs, RBPs and GWAS. Quantile-quantile (QQ) plots of *p* values for association with several traits and diseases, corresponding to sQTLs within the eCLIP peaks of RBFOX2, PRPF8, GTF2F1, SF3B4, GRWD1, PPIG, GEMIN5, CSTF2T and RBM15 (coloured dots), and the remaining sQTLs (black dots). The identity line is shown in red. Further information on *p* value calculation and other details of each GWAS are available at the corresponding publications, referenced in Methods. Source data are provided as a Source Data file.

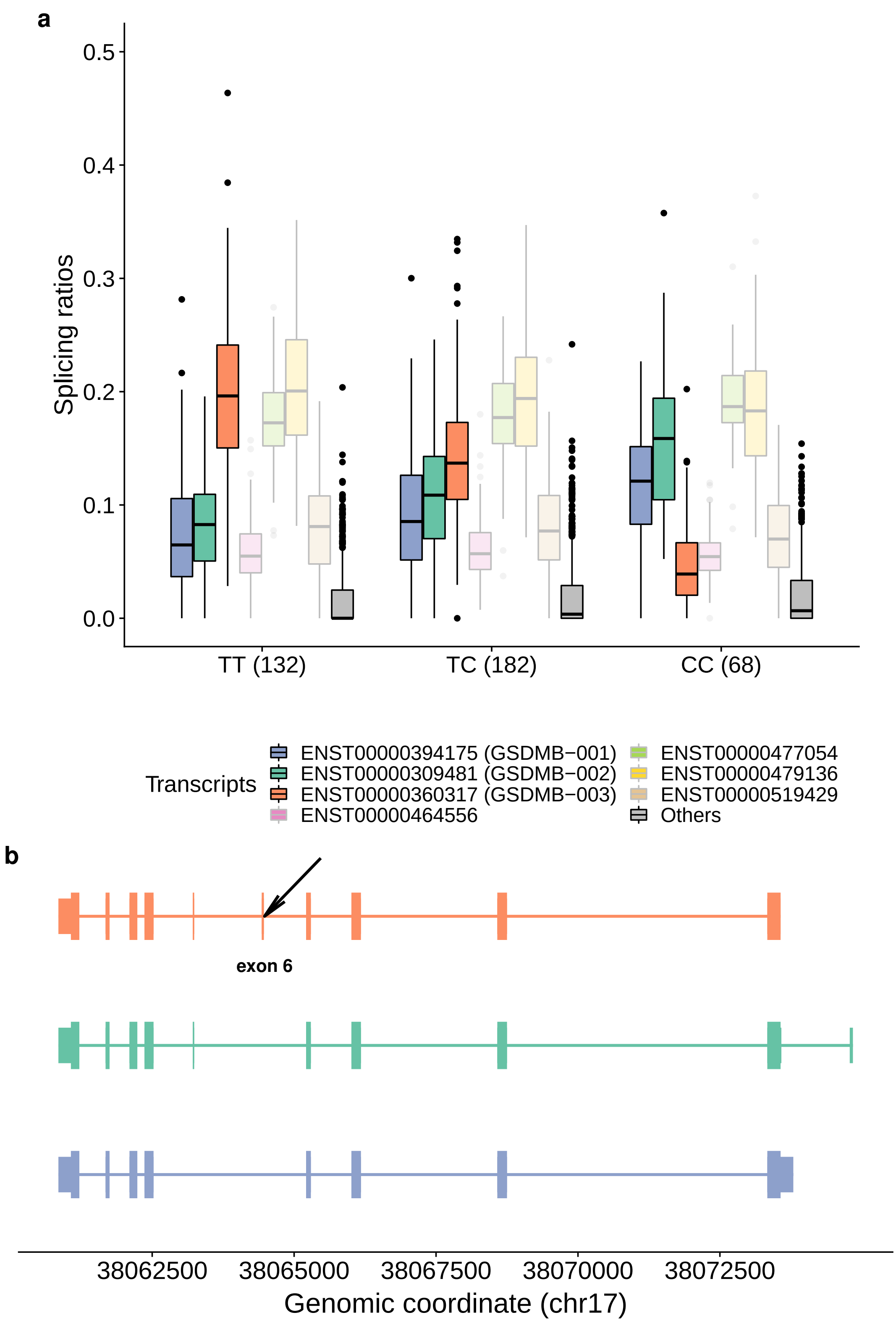

Supplementary Figure 22. rs11078928 and *GSDMB*. a) Relative abundances of the most expressed isoforms in lung from the gene *GSDMB* (chr17:38,060,848-38,076,107, reverse strand), for each genotype group at the rs11078928 locus (chr17:38,064,469, T/C), shown as boxplots. Solid and shaded colors correspond to protein coding and non-coding isoforms, respectively. The least abundant isoforms are grouped in Others. The number of individuals in each genotype group is shown between parentheses (total $n = 382$). Of all protein coding isoforms, *GSDMB-003* (red) is the most abundant in reference homozygous individuals at rs11078928 (TT). In contrast, in alternative homozygous individuals (CC), isoform *GSDMB-002* (green) is the most expressed. Isoform *GSDMB-001* (blue) shows a pattern analogous to *GSDMB-002* (green). Heterozygous individuals (TC) display an intermediate behaviour. The abundances of non-coding isoforms barely change with the genotype at rs11078928. In boxplots, the box represents the first to third quartiles and the median, and the whiskers indicate $\pm$ 1.5 $\times$ interquartile range (IQR). Source data are provided as a Source Data file. **b)** Exonic structure of the isoforms *GSDMB-001*, *GSDMB-002* and *GSDMB-003* and location of the rs11078928 SNP (marked with an arrow).

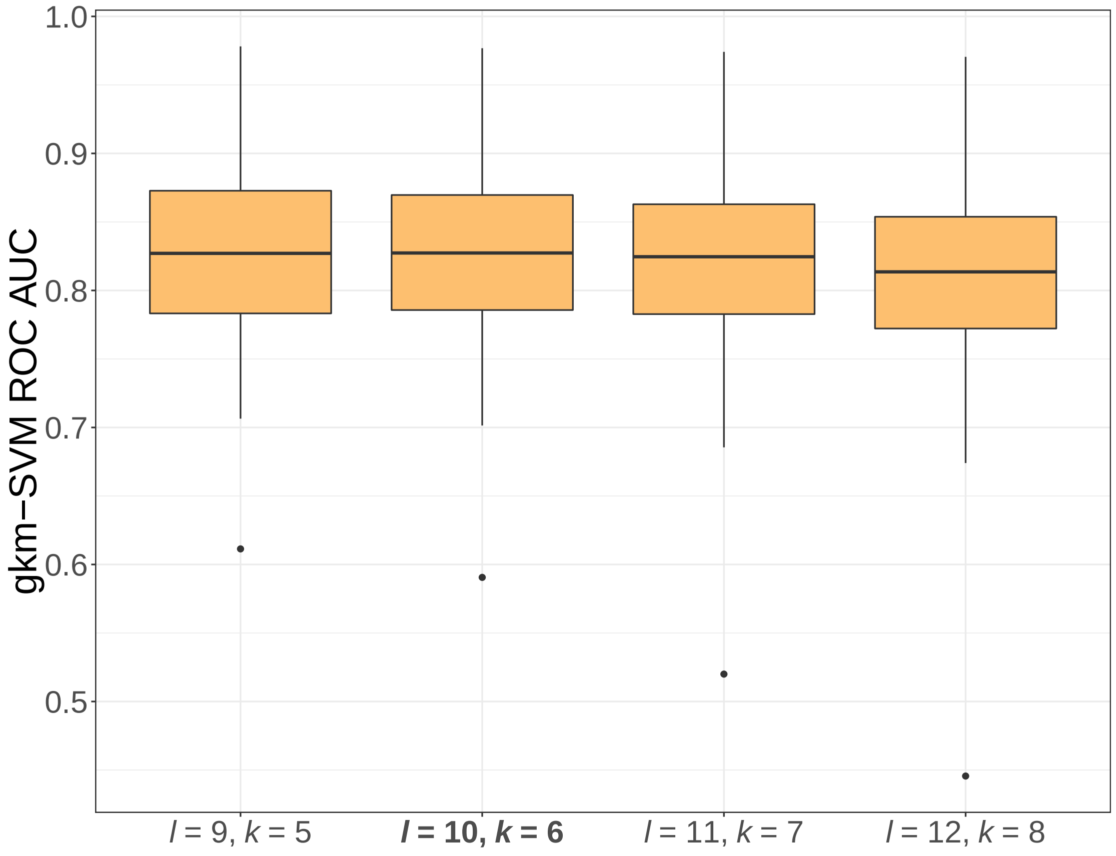

Supplementary Figure 23. **gkm-SVM classification performance for different parameter choices**. Comparison of the classification performance for $n = 113$ RBPs (measured as the mean cross-validation gkm-SVM ROC AUC per RBP), between different choices of the l (word length) and k (number of informative columns) parameters. Default values (l = 10, k = 6) are marked in bold. Data is shown as boxplots, where the box represents the first to third quartiles and the median, and the whiskers indicate $\pm$ 1.5 $\times$ interquartile range (IQR). Source data are provided as a Source Data file.

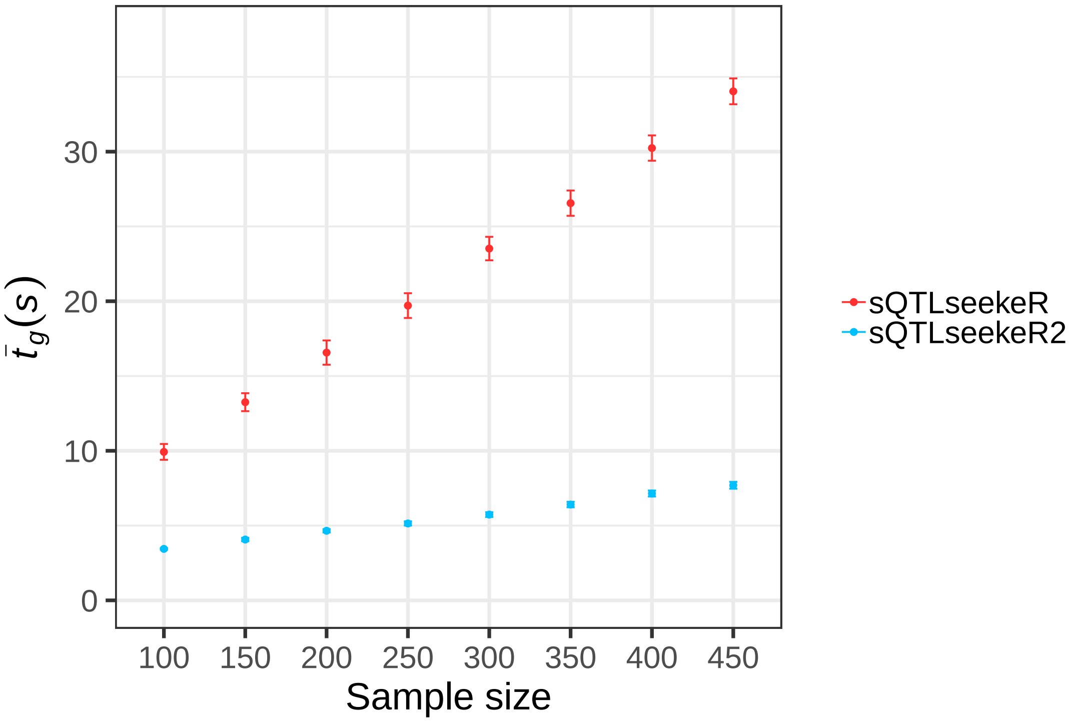

Supplementary Figure 24. **Running time of sQTLseekeR and sQTLseekeR2.** Dots represent the mean running time per gene ($\bar{t}_g$), in seconds, of sQTLseekeR (red) and sQTLseekeR2 (blue). Error bars represent the standard error of the mean (i.e. mean $\pm$ SEM). A nominal pass of both versions of the software was run on $n = 10$ sets of 100 randomly selected genes (on average 135 variants tested per gene), across a wide range of sample sizes, obtained by downsampling the GTEx Muscle Skeletal transcript expression dataset (the tissue with the largest sample size available). To make results comparable, we set common options to the same values, did not include covariates and did not perform additional filtering steps only available in sQTLseekeR2. We asked sQTLseekeR to perform 10^7 Monte Carlo generations. Source data are provided as a Source Data file.

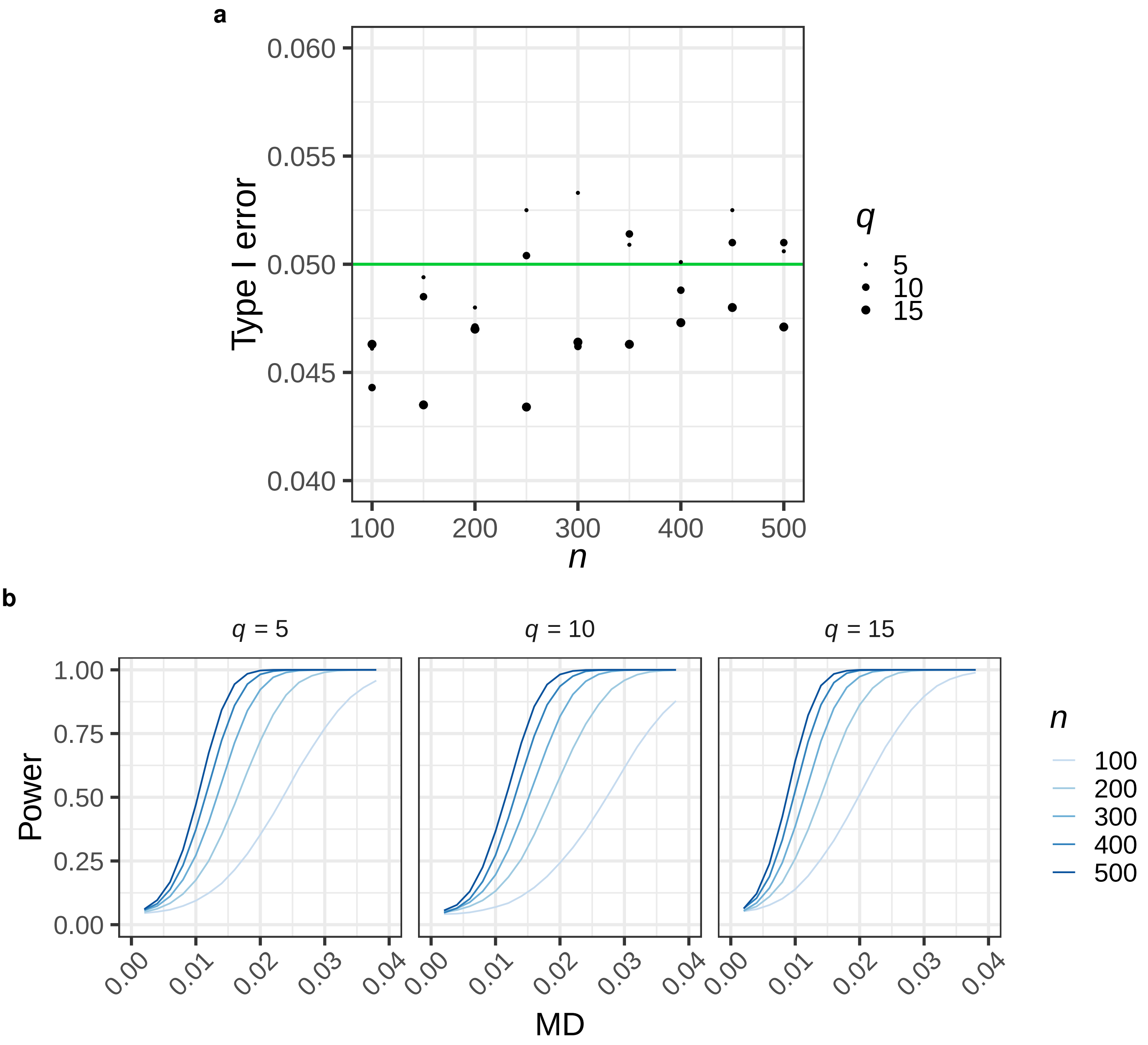

Supplementary Figure 25. Simulation study. a) Type I error of sQTLseekeR2 in simulated datasets with different sample size (n) and number isoforms studied (q). The horizontal green line marks the significance level selected (α = 0.05). **b)** Power of sQTLseekeR2 across simulated datasets with different values of n, q and sQTL effect sizes (MD values). See Supplementary Note 1 for details on the simulation. Source data for both a) and b) are provided as a Source Data file.

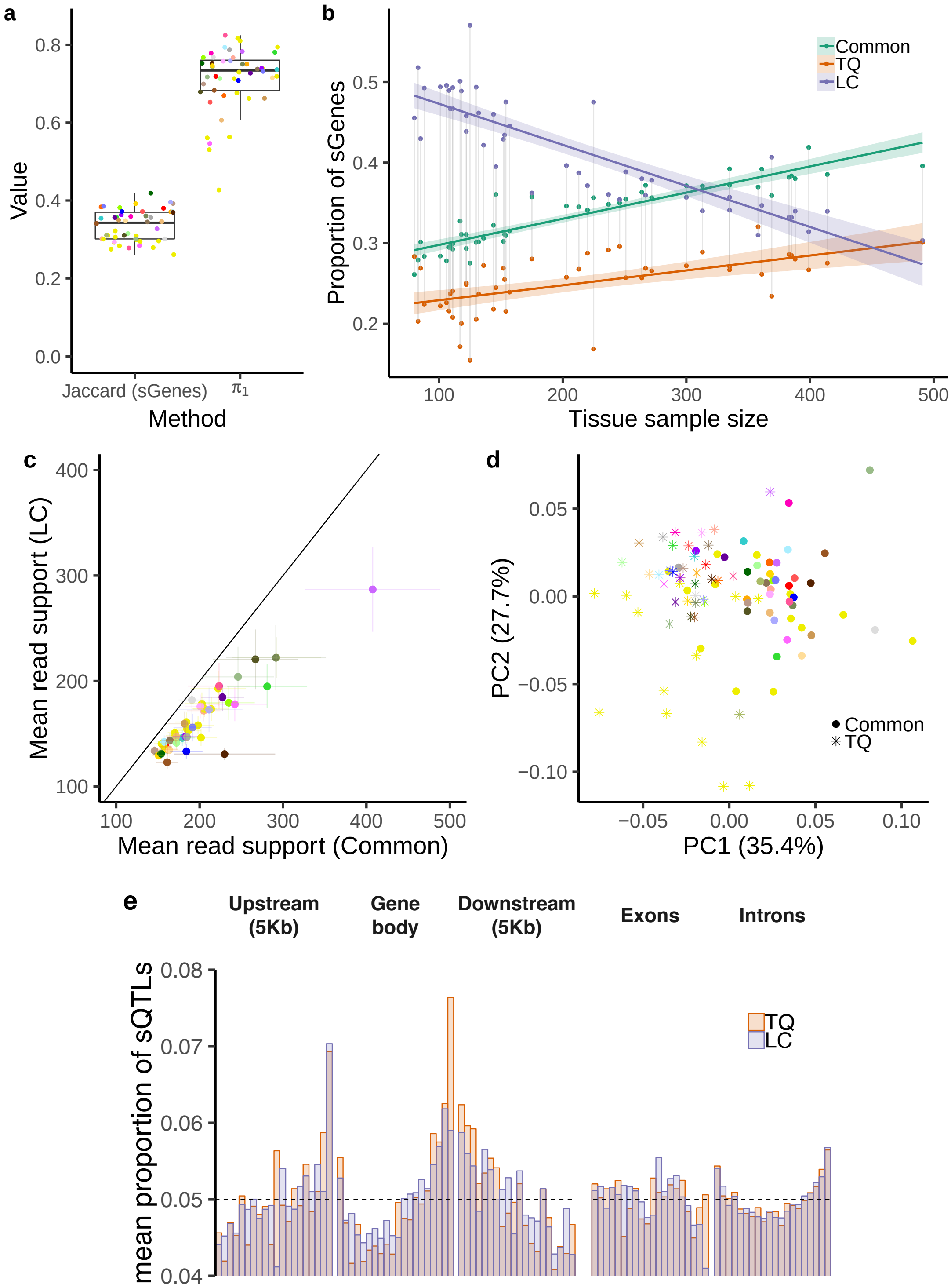

Supplementary Figure 26. sQTLseekeR2 with RSEM transcript quantifications and LeafCutter quantifications. a) Overlap between the sQTLs identified by sQTLseekeR2 using as input RSEM transcript quantifications and LeafCutter quantifications, measured at the level of sGenes (Jaccard index) and pairs sQTL-sGene (π_1) across $n = 48$ GTEx tissues. Data is shown as boxplots, where the box represents the first to third quartiles and the median, and the whiskers indicate $\pm$ 1.5 $\times$ interquartile range (IQR). **b)** Proportion of sGenes (y-axis) identified only with transcript quantifications (TQ), only with LeafCutter quantifications (LC), or with both (Common) *vs* tissue sample size (x-axis). Regression lines and 95% confidence intervals (coloured lines and areas, respectively) have been added to depict the trends. **c)** Dots representing the mean number of reads supporting a given intron cluster in each tissue for LC-exclusive (y-axis) *vs* Common (x-axis) sQTLs. Error bars represent the standard error of the mean (SEM) in each set of sQTLs (i.e. mean $\pm$ SEM). The identity line is shown in black. For visualization purposes, whole blood (x $=$ 640, y $=$ 403) is not displayed given its outlier behaviour. The total sample sizes (number of clusters) used for mean and SEM calculation can be found in the Source Data file. **d)** Representation in two dimensions, for each tissue, of the vector of proportions of the different types of AS events associated with Common and TC-exclusive sQTLs, obtained by PCA. Tissue color codes are shown in Supplementary Table 2. **e)** Comparison of the location of TQ-exclusive and LC-exclusive sQTLs. Source data for a) – e) are provided as a Source Data file.

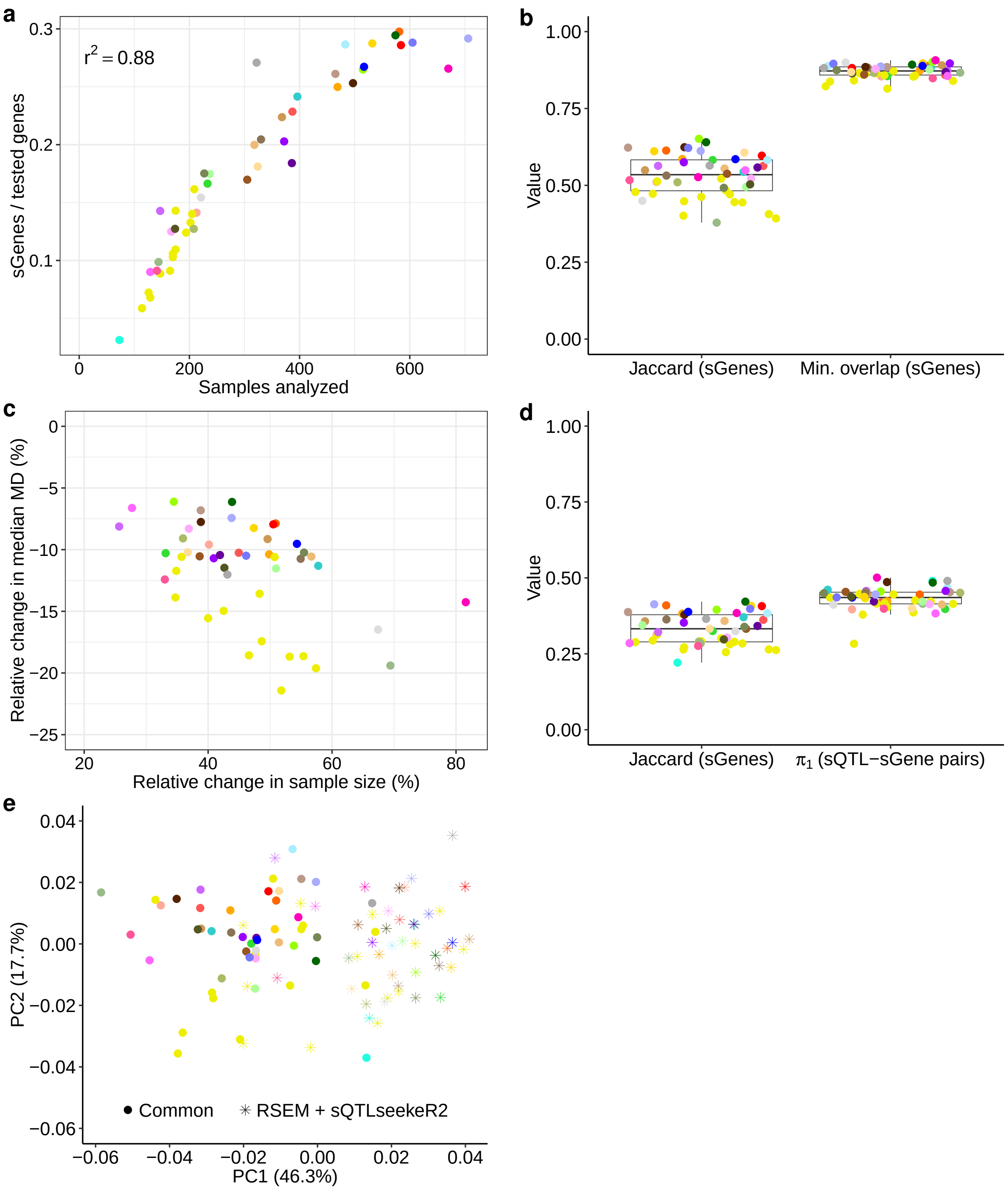

Supplementary Figure 27. sQTLs in GTEx V8. a) Proportion of sGenes (over tested genes) per tissue (y-axis) with respect to the tissue sample size (x-axis) identified in GTEx V8. Tissue color codes are shown in Supplementary Table 3. **b)** Overlap between the sGenes identified by sQTLseekeR2 in GTEx V7 and V8. Distribution of the Jaccard index (left) and the minimum overlap (that is, the number of common sGenes between V7 and V8 over the total number of sGenes identified in the smallest set, i.e. V7, right), computed per tissue ($n = 48$ tissues). To obtain these metrics only the genes tested in both V7 and V8 are considered. **c)** Relative change in median sQTL effect size (median MD value) between V8 and V7, per tissue (y-axis), *vs* the relative change in tissue sample size (x-axis). **d)** Overlap between the sQTLs identified by RSEM + sQTLseekeR2 and the ones obtained using LeafCutter + FastQTL by the GTEx Consortium, measured at the level of sGenes (Jaccard index) and pairs sQTL-sGene (π_1) across $n = 48$ tissues. Note that the overlap was evaluated considering the variant-gene-tissue trios tested in both runs. Both in b) and d), data is shown as boxplots, where the box represents the first to third quartiles and the median, and the whiskers indicate $\pm$ 1.5 $\times$ interquartile range (IQR). **e)** Representation in two dimensions, for each tissue, of the vector of proportions of the different types of AS events associated with the sQTLs identified only by RSEM + sQTLseekeR2 and in common with the GTEx Consortium (obtained by PCA). Source data for a) – e) are provided as a Source Data file.

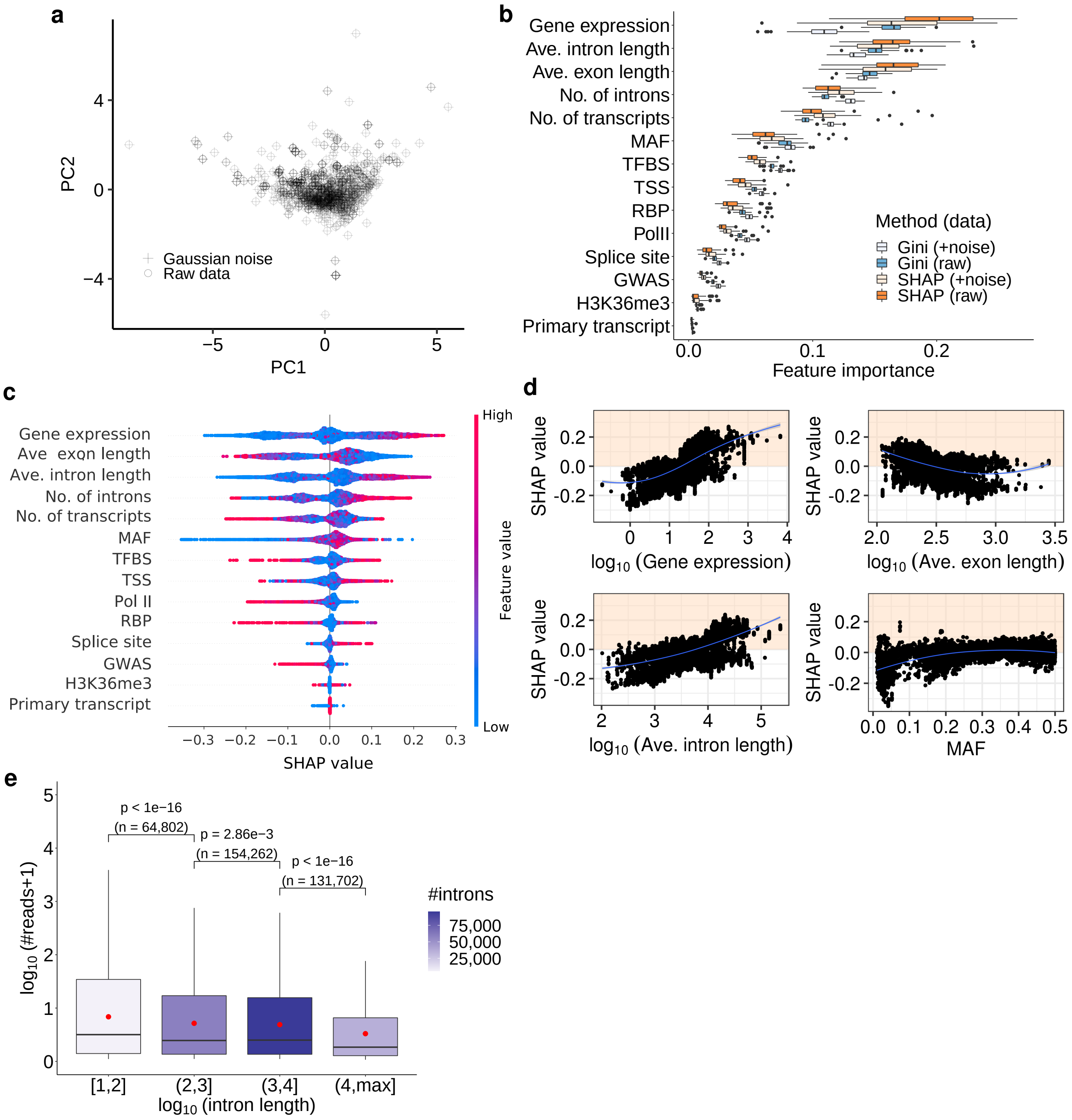

Supplementary Figure 28. Characterization of approach-exclusive sQTLs in GTEx V8. a) PCA of the matrix of sQTL-sGene associations (rows) by gene features (columns) corresponding to esophagus (gastroesophageal junction), before and after the addition of gaussian noise. **b)** Feature importances estimated via the mean decrease in Gini index and the mean absolute SHAP value, before and after the addition of gaussian noise to the gene features (each boxplot corresponds to $n = 49$ tissues). **c)** SHAP values representing, for each feature, the contribution of each sQTL-sGene association in esophagus (gastroesophageal junction) to classification in the LCFQ or in the RMSQ class (positive and negative values, respectively). Features are sorted according to their importance, and feature values are color-coded. **d)** For the features displaying correlation between high (low) values and classification in one of the classes in c), we represented SHAP values (x-axis) *vs* the actual feature values (y-axis). A polynomial was fitted to the points using local fitting (LOESS) in order to describe the trend (fit in blue, 95% confidence interval in grey). Positive SHAP values (coloured region) correspond to observations that contribute to classification in the LCFQ class, while negative SHAP values (white region) correspond to observations that contribute to classification in the RMSQ class. **e)** Distribution of the average number of reads supporting an intron with respect to intron length in esophagus (gastroesophageal junction, data from our analyses in Supplementary Note 2). Red dots represent the mean. The number of introns in each group is color-coded. Two-sided Wilcoxon Rank-Sum test p values and sample sizes are shown for each pairwise comparison. In b) and e), data is shown as boxplots, where the box represents the first to third quartiles and the median, and the whiskers indicate $\pm$ 1.5 $\times$ interquartile range (IQR). Source data for a) – e) are provided as a Source Data file.

Tissue	Samples	Variants	Genes	Associations	sQTLs	sGenes
Adipose - Subcutaneous	385	1,407,668	10,425	58,152	57,347	2,206
Adipose - Visceral (Omentum)	313	1,263,199	10,454	44,103	43,410	1,794
Adrenal Gland	175	986,571	10,272	26,289	25,863	1,164
Artery - Aorta	267	1,205,692	10,365	42,011	41,378	1,609
Artery - Coronary	152	958,484	10,511	18,877	18,637	887
Artery - Tibial	388	1,379,506	10,114	54,533	53,769	1,994
Brain - Amygdala	88	719,074	10,075	5,943	5,884	314
Brain - Anterior cingulate cortex (BA24)	109	869,601	10,337	9,559	9,446	477
Brain - Caudate (basal ganglia)	144	991,073	10,382	14,479	14,195	695
Brain - Cerebellar Hemisphere	125	916,855	10,729	15,582	15,249	731
Brain - Cerebellum	154	1,041,020	10,807	25,631	25,202	1,017
Brain - Cortex	136	1,017,886	10,560	16,007	15,799	778
Brain - Frontal Cortex (BA9)	118	957,206	10,633	12,447	12,316	613
Brain - Hippocampus	111	853,881	10,300	8,228	8,148	422
Brain - Hypothalamus	108	885,872	10,589	10,013	9,914	490
Brain - Nucleus accumbens (basal ganglia)	130	957,657	10,493	12,775	12,661	604
Brain - Putamen (basal ganglia)	111	801,461	9,872	11,656	11,478	502
Brain - Spinal cord (cervical c-1)	83	669,619	10,327	6,942	6,873	334
Brain - Substantia nigra	80	656,998	10,141	5,037	4,970	280
Breast - Mammary Tissue	251	1,175,934	10,475	39,953	39,299	1,558
Cells - EBV-transformed lymphocytes	117	590,120	8,421	16,282	16,102	731
Cells - Transformed fibroblasts	300	1,127,243	9,464	43,890	43,293	1,652
Colon - Sigmoid	203	1,102,171	10,577	32,983	32,563	1,321
Colon - Transverse	246	1,115,378	10,321	34,833	34,415	1,359
Esophagus - Gastroesophageal Junction	213	1,101,011	10,480	32,313	31,778	1,288
Esophagus - Mucosa	358	1,298,073	10,568	48,733	47,941	1,901
Esophagus - Muscularis	335	1,358,213	10,477	50,653	49,826	1,920
Heart - Atrial Appendage	264	1,137,415	9,958	33,263	32,723	1,284
Heart - Left Ventricle	272	984,193	8,991	24,583	24,122	991
Liver	153	738,737	9,282	12,635	12,471	630
Lung	383	1,497,417	11,309	56,078	55,251	2,159
Minor Salivary Gland	85	625,502	10,330	6,782	6,655	442
Muscle - Skeletal	491	1,182,336	8,835	49,633	48,982	1,810
Nerve - Tibial	361	1,501,244	11,075	58,707	57,990	2,224
Ovary	122	854,281	10,433	16,883	16,714	748
Pancreas	220	941,010	9,505	24,737	24,545	1,044
Pituitary	157	1,093,565	11,282	24,929	24,647	1,083
Prostate	132	936,634	11,030	15,593	15,393	809
Skin - Not Sun Exposed (Suprapubic)	335	1,303,847	10,730	49,220	48,263	1,899
Skin - Sun Exposed (Lower leg)	414	1,440,899	10,836	60,061	58,953	2,232
Small Intestine - Terminal Ileum	122	811,821	10,648	15,218	15,089	726
Spleen	146	894,283	10,532	22,998	22,781	1,043
Stomach	237	1,044,155	10,020	27,184	26,905	1,184
Testis	225	1,513,533	12,687	58,402	57,589	2,111
Thyroid	399	1,523,381	11,133	64,120	62,919	2,434
Uterus	101	781,865	10,720	12,755	12,685	635
Vagina	106	790,813	10,768	12,140	11,923	578
Whole Blood	369	633,825	6,327	21,566	21,318	920
**Average**	214	1,034,130	10,304	28,446	28,034	1,158
**Total**	10,294	3,588,609	16,010	216,961	210,485	6,963

Supplementary Table 1. Number of samples, variants and genes tested; variant-gene associations, sQTLs and sGenes identified across tissues (FDR < 0.05) in GTEx V7.

Tissue	Samples	Variants	Genes	Associations	sQTLs	sGenes
Adipose - Subcutaneous	385	758,333	5,975	44,148	41,083	1,625
Adipose - Visceral (Omentum)	313	645,188	5,801	32,899	30,526	1,340
Adrenal Gland	175	357,993	4,302	19,987	18,129	842
Artery - Aorta	267	580,788	5,284	31,441	29,361	1,209
Artery - Coronary	152	431,275	5,266	18,085	17,140	824
Artery - Tibial	388	711,276	5,499	41,560	38,451	1,472
Brain - Amygdala	88	182,912	3,261	5,876	5,371	298
Brain - Anterior cingulate cortex (BA24)	109	243,497	3,642	9,462	8,682	437
Brain - Caudate (basal ganglia)	144	304,661	3,919	12,104	11,104	571
Brain - Cerebellar Hemisphere	125	405,495	5,232	23,494	19,834	952
Brain - Cerebellum	154	461,286	5,327	30,505	26,398	1,141
Brain - Cortex	136	364,844	4,398	15,213	13,878	667
Brain - Frontal Cortex (BA9)	118	311,326	4,025	11,854	10,918	558
Brain - Hippocampus	111	218,704	3,402	7,940	7,250	385
Brain - Hypothalamus	108	254,314	3,938	9,055	8,315	456
Brain - Nucleus accumbens (basal ganglia)	130	273,022	3,794	11,931	10,988	571
Brain - Putamen (basal ganglia)	111	214,012	3,318	8,450	7,645	436
Brain - Spinal cord (cervical c-1)	83	206,183	3,731	6,777	6,415	363
Brain - Substantia nigra	80	166,463	3,286	4,716	4,356	257
Breast - Mammary Tissue	251	610,844	5,895	35,075	32,411	1,371
Cells - EBV-transformed lymphocytes	117	315,504	4,442	22,263	21,048	933
Cells - Transformed fibroblasts	300	589,043	4,990	33,247	30,955	1,218
Colon - Sigmoid	203	462,574	5,134	27,428	25,914	1,108
Colon - Transverse	246	456,973	4,909	21,475	19,961	969
Esophagus - Gastroesophageal Junction	213	441,111	4,824	25,416	23,862	1,057
Esophagus - Mucosa	358	477,846	4,524	26,603	25,044	1,115
Esophagus - Muscularis	335	565,549	4,914	36,681	33,985	1,353
Heart - Atrial Appendage	264	478,621	4,480	22,583	21,026	928
Heart - Left Ventricle	272	270,243	2,900	14,371	13,401	600
Liver	153	190,231	2,963	11,037	10,085	523
Lung	383	715,284	6,003	36,182	34,324	1,527
Minor Salivary Gland	85	206,445	4,097	7,132	6,801	416
Muscle - Skeletal	491	516,129	4,100	27,539	26,127	1,089
Nerve - Tibial	361	753,162	6,180	48,704	45,256	1,790
Ovary	122	355,980	5,204	17,135	16,103	778
Pancreas	220	281,415	3,336	15,084	13,873	703
Pituitary	157	393,827	5,170	26,231	24,316	1,062
Prostate	132	338,794	5,044	16,227	15,103	819
Skin - Not Sun Exposed (Suprapubic)	335	617,127	5,628	36,497	34,260	1,485
Skin - Sun Exposed (Lower leg)	414	685,673	5,732	40,761	38,375	1,632
Small Intestine - Terminal Ileum	122	311,399	4,876	12,592	11,946	642
Spleen	146	328,298	4,525	19,206	18,026	895
Stomach	237	369,489	4,150	15,420	14,494	777
Testis	225	789,414	7,769	89,009	83,724	2,739
Thyroid	399	735,487	6,029	48,250	44,783	1,807
Uterus	101	334,515	5,323	14,358	13,405	711
Vagina	106	286,538	4,763	12,357	11,497	624
Whole Blood	369	295,509	3,020	18,687	17,085	732
**Average**	214	422,179	4,673	23,397	21,730	954
**Total**	10,294	2,507,537	14,045	286,349	263,827	7,471

Supplementary Table 2. Number of samples, variants and genes tested; variant-gene associations, sQTLs and sGenes identified across tissues (FDR < 0.05) in GTEx V7 using the vector of intron excision ratios of an intron cluster obtained by LeafCutter as splicing phenotype.

Tissue	Samples	Variants	Genes	Associations	sQTLs	sGenes
Adipose - Subcutaneous	581	1,849,202	10,494	101,905	100,331	2,990
Adipose - Visceral (Omentum)	469	1,759,158	10,604	79,846	78,490	2,487
Adrenal Gland	233	1,248,945	10,393	43,530	42,912	1,555
Artery - Aorta	387	1,634,096	10,520	72,071	70,843	2,272
Artery - Coronary	213	1,269,445	10,646	36,838	36,244	1,367
Artery - Tibial	584	1,808,194	10,208	92,154	90,652	2,720
Brain - Amygdala	129	997,280	10,155	14,306	14,141	580
Brain - Anterior cingulate cortex (BA24)	147	1,135,042	10,448	17,973	17,782	754
Brain - Caudate (basal ganglia)	194	1,269,547	10,425	28,549	28,010	1,052
Brain - Cerebellar Hemisphere	175	1,205,236	10,814	33,739	33,013	1,236
Brain - Cerebellum	209	1,355,112	10,902	44,581	43,584	1,581
Brain - Cortex	205	1,414,047	10,693	36,624	36,217	1,321
Brain - Frontal Cortex (BA9)	175	1,303,066	10,749	25,322	25,105	996
Brain - Hippocampus	165	1,139,777	10,250	20,053	19,871	756
Brain - Hypothalamus	170	1,233,848	10,594	24,432	24,157	883
Brain - Nucleus accumbens (basal ganglia)	202	1,303,678	10,457	28,187	27,832	1,042
Brain - Putamen (basal ganglia)	170	1,096,223	9,910	22,676	22,358	891
Brain - Spinal cord (cervical c-1)	126	971,511	10,456	17,001	16,865	669
Brain - Substantia nigra	114	890,437	10,080	11,457	11,340	515
Breast - Mammary Tissue	396	1,651,603	10,583	77,202	76,009	2,381
Cells - EBV-transformed lymphocytes	483	1,521,528	9,381	79,240	77,956	2,339
Cells - Transformed fibroblasts	147	743,422	8,435	28,101	27,708	1,045
Colon - Sigmoid	318	1,564,926	10,720	62,501	61,303	1,969
Colon - Transverse	368	1,555,065	10,466	65,543	64,518	2,082
Esophagus - Gastroesophageal Junction	330	1,566,911	10,625	63,217	62,335	2,011
Esophagus - Mucosa	497	1,700,995	10,651	78,535	77,340	2,499
Esophagus - Muscularis	465	1,811,046	10,620	83,766	82,229	2,537
Heart - Atrial Appendage	372	1,523,837	10,069	58,573	57,816	1,896
Heart - Left Ventricle	386	1,284,915	9,072	44,520	43,827	1,436
Kidney - Cortex	73	540,123	9,741	6,701	6,622	336
Liver	208	948,583	9,366	26,424	26,090	1,003
Lung	515	1,937,020	11,391	92,364	90,862	2,803
Minor Salivary Gland	144	1,017,962	10,674	22,910	22,486	966
Muscle - Skeletal	706	1,482,994	8,910	84,962	83,542	2,440
Nerve - Tibial	532	2,007,715	11,196	104,184	102,300	3,075
Ovary	167	1,101,628	10,571	32,306	31,812	1,176
Pancreas	305	1,213,421	9,490	44,223	43,518	1,549
Pituitary	237	1,490,248	11,386	54,580	53,717	1,795
Prostate	221	1,385,232	11,198	42,801	42,063	1,556
Skin - Not Sun Exposed (Suprapubic)	517	1,785,930	10,836	88,168	86,855	2,710
Skin - Sun Exposed (Lower leg)	605	1,864,176	10,901	99,943	98,230	3,032
Small Intestine - Terminal Ileum	174	1,118,322	10,838	29,823	29,563	1,179
Spleen	227	1,266,159	10,739	48,529	47,709	1,745
Stomach	324	1,387,919	10,133	45,025	44,442	1,552
Testis	322	2,033,706	12,712	111,059	109,218	3,087
Thyroid	574	1,992,634	11,235	107,543	105,641	3,174
Uterus	129	982,504	10,823	22,514	22,209	908
Vagina	141	1,011,962	10,940	22,196	21,866	870
Whole Blood	670	799,480	6,132	44,875	44,132	1,476
**Average**	310	1,370,935	10,380	51,501	50,687	1,679
**Total**	15,201	4,074,385	16,202	356,682	344,211	9,051

Supplementary Table 3. Number of samples, variants and genes tested; variant-gene associations, sQTLs and sGenes identified across tissues (FDR $<$ 0.05) in GTEx V8. Note that Cells - Transformed fibroblasts has been modified to Cells - Cultured fibroblasts with respect to GTEx V7.

Supplementary Notes

Supplementary Note 1

sQTLseekeR and sQTLseekeR2

sQTLseekeR[1] identifies genetic variants that are associated with changes in the relative abundances of a gene's transcript isoforms (i.e. splicing ratios). The splicing ratio of isoform i in individual j is $f_{ij} = x_{ij}/\sum_{i=1}^{q} x_{ij}$, where x_{ij} is the expression of isoform i in individual j and q is the number of isoforms of the gene. Note that splicing ratios configure a multivariate phenotype, with as many values as there are transcripts for a given gene. sQTLseekeR uses the Hellinger distance between splicing ratios to estimate their variability across individuals. The Hellinger distance between the splicing ratios of individuals j and k is given by:

$$d_H(j,k) = \sqrt{\sum_{i=1}^{q}(\sqrt{f_{ij}} - \sqrt{f_{ik}})^2} \tag{1}$$

For a given gene and genetic variant, sQTLseekeR compares the variability of the gene's splicing ratios, calculated as a sum of squares, within and between genotypes at the variant (i.e. 0, 1, 2). The comparison is performed computing a pseudo F score as defined by Anderson[2]:

$$\tilde{\mathrm{F}} = \frac{SS_B}{SS_W} = \frac{SS_T - SS_W}{SS_W} \tag{2}$$

where SS_T is the total variability, SS_B is the variability between genotypes, and SS_W is the variability within genotypes. If N is the total number of individuals,

$$SS_T = \frac{1}{N}\sum_{j=1}^{N-1}\sum_{k=j+1}^{N} d_H^2(j,k) \tag{3}$$

where $d_H^2(j,k)$ is the squared Hellinger distance between the splicing ratios of individuals j and k, and

$$SS_W = \sum_{g=1}^{p}\frac{1}{n_g}\sum_{j=1}^{N-1}\sum_{k=j+1}^{N} d_H^2(j,k)\epsilon_{g,j,k} \tag{4}$$

where p is the number of genotypes at the variant, n_g the number of individuals with genotype g, and $\epsilon_{g,j,k} = 1$ if individuals j and k have genotype g at the variant, otherwise $\epsilon_{g,j,k} = 0$.

To assess significance, sQTLseekeR relies on the asymptotic distribution of pseudo F scores. Anderson derived the asymptotic distribution for the numerator of the pseudo F score, which is a linear combination of independent χ^2 variables with n_g-1 degrees of freedom, where the coefficients are derived from the matrix built upon the distances between every pair of individuals[3]. For any set of permutations of a given data set, the sum of squares of the numerator is a monotonic function of the pseudo F score, thus the permutation *p* values computed on the numerator and on the pseudo F score are equivalent. Essentially, this approach is a nonparametric analogue to multivariate analysis of variance (MANOVA). sQTLseekeR2, available at `https://github.com/guigolab/sQTLseekeR2`, is a completely rewritten, largely enhanced version of sQTLseekeR

(see below), although the statistical framework implemented remains the same. Notably, this framework is not necessarily restricted to the analysis of relative transcript abundances, and can be applied to other multivariate AS phenotypes (see also Supplementary Note 2).

Enhancements in sQTLseekeR2

p value computation

To approximate the asymptotic distribution of the test statistic, sQTLseekeR relied on the Monte Carlo generation of χ^2 distributed values. Therefore, the smallest achievable *p* value was $1/(M+1)$, where M was the number of Monte Carlo generations. Due to memory and time limitations, in practice M takes values up to 10^7. In contrast, sQTLseekeR2 relies on Davies algorithm[4] to perform this task. The algorithm allows to approximate the CDF of the asymptotic distribution of the test statistic with high accuracy through the numerical inversion of the characteristic function. This has considerably decreased the *p* value precision limit (up to 10^{-14}) and speeded up the *p* value computation.

Correction for potential confounders

sQTLseekeR2 allows the incorporation of potential technical or biological confounders (either numerical or categorical) as covariates to be regressed out from the splicing ratios before testing for association with the genotype.

Multiple testing correction scheme

In *cis* sQTL mapping, all possible variant-gene pairs in *cis* are tested for association. This implies two multiple-testing levels: i) multiple variants are tested per gene, and ii) multiple genes are tested genome-wide. To account for (i), sQTLseekeR2 implements a permutation scheme that empirically characterizes, for each gene, the distribution of nominal *p* values expected under the null hypothesis of no association[5]. This null distribution is then modeled using a beta distribution as in FastQTL[6]. In short, the minimal nominal *p* value (p_{min}) per gene is used as the test statistic. Permutations are performed by randomizing sample labels for the splicing ratios. By default, a maximum of 1,000 permutations is performed, with a stopping criteria of having at least 100 permuted p'_{min} values lower than the nominal p_{min}. A maximum likelihood-fitted beta distribution from the permutations is used to compute gene-level empirical *p* values. To account for (ii) and identify sGenes, sQTLseekeR2 uses false discovery rate (FDR). Due to the computational burden of the permutations, a simpler approach (originally in sQTLseekeR) based on performing FDR on pooled nominal *p* values is also available. This can be further enhanced in sQTLseekeR2 by clustering variants in high linkage disequilibrium (see section *Linkage disequilibrium-based variant clustering*).

To identify all significant variant-gene pairs, sQTLseekeR2 implements a procedure identical to the one depicted in 7 for expression QTLs: i) it defines a genome-wide empirical *p* value threshold, p_t, as the gene-level

p value closest to the 0.05 FDR threshold; ii) for each gene, it computes a nominal *p* value threshold based on the beta distribution model of the minimum *p* value distribution $f(p_{min})$ (obtained from the permutations for this gene, see above), as $F^{-1}(p_t)$ (where F^{-1} is the inverse cumulative distribution); iii) for each gene, variants with a nominal *p* value below the gene-level threshold are considered significant.

Linkage disequilibrium-based variant clustering

A substantial fraction of the variants tested in *cis* (up to 50% for a window of 5 Kb around the gene) are in high ($r^2 \geq$ 0.8) or even complete ($r^2 =$ 1) linkage disequilibrium (LD). sQTLseekeR2 allows clustering variants above a user-defined LD threshold, so that only a representative of each cluster is tested for association. This reduces the number of dependent tests and therefore the running time and stringency of the multiple testing correction. It can be combined with simple FDR as a fast, suboptimal alternative to permutations for multiple testing correction.

Reduced running time

The more efficient *p* value computation, along with other minor improvements to speed up the preprocessing steps, allowed to achieve a substantial reduction (between three and four times) of the running time of a nominal pass of sQTLseekeR2 with respect to sQTLseekeR (Supplementary Figure 24).

Simulation study

To study the type I error of sQTLseekeR2, we simulated the splicing ratios of 10,000 genes with q transcript isoforms in n individuals, under the null hypothesis of no association with genetic variants. We evaluated different scenarios modifying the values of $q \in \{5, 10, 15\}$ (selected matching the distribution of the number of splicing isoforms per gene considered in our sQTL analysis) and $n \in$ [100, 500] (selected matching the tissue sample sizes in GTEx V7). We simulated one SNP per gene, the probability of an individual belonging to each genotype group (i.e. 0, 1, 2) being 0.6, 0.3 and 0.1, respectively. Splicing ratios were simulated as vectors of proportions in the $q-1$ simplex with mean $\mathbf{c} = \mathbf{q}^{-1}$ (i.e. the center of the simplex) for all genotype groups. To generate observations in the simplex with certain variability around $\mathbf{c}$, we performed q random displacements of size $\sim \mathcal{N}(0, \sigma)$ from $\mathbf{c}$ towards the simplex vertices. σ was selected so that the mean standard deviation of splicing ratios was constant and approximately equal to 0.03 across different values of q, ensuring that the resulting vectors of proportions were elements of the simplex. We estimated type I error for each combination of q and n as the fraction of tests (out of 10,000) found significant at $\alpha =$ 0.05. Our results show that sQTLseekeR2 presents overall a controlled type I error rate (Supplementary Figure 25a).

To study power, we simulated splicing ratios under the alternative hypothesis. First, we generated vectors of proportions in the simplex as above. Then we incremented the splicing ratio of the first transcript isoform in an amount Δ (decreasing accordingly the splicing ratios of the other transcript isoforms in $\Delta/(q-1)$) for individuals with genotype 0 at the SNP of interest. We did the opposite for individuals with genotype 1 at this

SNP. We explored values of Δ in the range (0, 0.02]. In practice, $\Delta =$ MD/2, where MD is the sQTL effect size (see MD definition in Methods). To estimate power, for each combination of n, q and MD, we computed the fraction of tests significant at $\alpha =$ 0.05. Our results show that sQTLseekeR2 presents a very large power to detect differences in splicing ratios across genotype groups (Supplementary Figure 25b), even when effect sizes are very small (e.g. with $q =$ 10 and $n =$ 300, MD $=$ 0.02 is detected with power 0.92).

Nextflow pipeline

sQTL mapping in large sequencing projects such as GTEx, with a large number of samples, a high density genotyping and millions of statistical tests, requires a parallelization strategy to absorb such a computational load in reasonable running times. However, any given implementation of an sQTL mapping pipeline may be restricted to a particular computing platform, hindering reproducibility, portability and scalability. Nextflow is a domain-specific language (DSL) for parallel computational pipelines[8]. It allows to execute a pipeline on multiple platforms without changes, and supports container technologies such as Docker (`https://www.docker.com`) or Singularity (`https://singularity.lbl.gov`), ensuring reproducibility. Taking advantage of these features, we have developed an sQTL mapping pipeline using sQTLseekeR2, Nextflow and Docker, in order to map sQTLs in GTEx. Our pipeline, named sqtlseeker2-nf, is available at `https://github.com/guigolab/sqtlseeker2-nf`.

Supplementary Note 2

LeafCutter

A number of studies have recently employed LeafCutter to identify sQTLs[9–11]. LeafCutter[12] is a method to quantify alternative splicing (AS) from short-read RNA-seq data. It uses split-mapped reads to identify alternatively-excised introns and groups them into clusters. Then, intron usage can be expressed as proportions (i.e. intron excision ratios: the number of reads supporting an intron over the total number of reads supporting the cluster to which the intron has been assigned). To test for association with genetic variants, individual intron excision ratios are often modeled using univariate linear regression (e.g. as implemented in FastQTL[6]).

The vector of intron excision ratios obtained by LeafCutter, however, can also be modeled as a multivariate phenotype, and therefore it can be employed as input for sQTLseekeR2 to identify sQTLs. Actually, multivariate modeling through a Dirichlet-multinomial generalized linear model (GLM) is available within LeafCutter, but only for differential splicing analyses. Given the popularity of LeafCutter, we have run sqtlseeker2-nf with LeafCutter quantifications in GTEx V7, and compared the resulting sQTLs with the set obtained using RSEM transcript quantifications.

LeafCutter AS quantification and sQTL mapping

We quantified AS based on the intron excision phenotypes defined by LeafCutter as follows: first, we used the `bam2junc.sh` script to quantify intron usage, and then we employed the `leafcutter_cluster.py` script (with options `--min_clu_reads 30 --min_clu_ratio 0.001 --max_intron_len 500000`) to define intron clusters. Both scripts are provided with LeafCutter software. To map LeafCutter clusters to genes, we employed the `map_clusters_to_genes.R` script available from `https://github.com/broadinstitute/gtex-pipeline`, with exon coordinates derived from GENCODE v19.

The resulting `*_perind_numers.counts.gz` files were modified to include two ID columns: i) intron:cluster:gene and ii) cluster:gene, and used as input for sqtlseeker2-nf, in place of tissue transcript expression files. Note that these columns replace the transcript and gene ID columns required when using transcript quantifications. By setting `--min_transcript_expr 5 --min_gene_expr 30` in sQTLseekeR2, we considered clusters supported by $\geq$ 30 reads in at least 80% of the samples (samples with lower cluster read support were removed from the analysis of the cluster), with at least two introns and a minimum intron read count of five (introns with lower read support in all samples were removed). The remaining settings (covariate correction, variant filtering, FDR threshold, etc.) were identical to the run using transcript quantifications (see section *sQTL mapping* in Methods). Note that for each cluster, the *cis* window was defined as the corresponding gene body plus 5 Kb upstream and downstream the gene boundaries. In total, 2,507,537 variants and 14,045 genes (13,347 protein coding, 698 lincRNA) were analyzed. At a 0.05 false discovery rate (FDR), we found a total of 263,827 *cis* sQTLs affecting 7,471 sGenes (7,046 protein coding genes and 425 lincRNAs) (Supplementary Table 2).

Comparison between sQTLs identified using RSEM transcript quantifications and LeafCutter intron excision ratios

To obtain comparable results, genes that were not tested in both runs were filtered out, and the multiple testing correction (process `permuted_mtc` in sqtlseeker2-nf) was repeated on the set of common genes. In addition, the selection of the same *cis* window and parameters regarding variant filtering in both runs ensured that the set of variants tested was virtually identical. We compared the pairs sQTL-sGene identified by i) both approaches (i.e. common sQTLs), ii) LeafCutter quantifications only (i.e. LC-exclusive sQTLs), and iii) transcript quantifications only (i.e. TQ-exclusive sQTLs). Note that in the case of LeafCutter, we considered that an sQTL affects a given gene if it is associated with changes in intron excision ratios of at least one of the intron clusters assigned to this gene.

Overall, we observed a moderate overlap between the sQTL sets identified by the two approaches (median Jaccard index of sGenes across tissues 0.34, median π_1 of TQ in LC sQTL-sGene associations across tissues 0.73, Supplementary Figure 26a). The number of LC-exclusive sQTLs was consistently larger than the number of TQ-exclusive sQTLs. This could be explained by the fact that LeafCutter is able to detect novel introns, whereas transcript quantification relies only on GENCODE v19 annotation. Indeed, 44% (median across tissues) of the most-changing introns within intron clusters associated with LC-exclusive sQTLs are novel. However, we observed that the proportion of LC-exclusive sGenes decreased with the tissue sample size, while the proportion of common and TQ-exclusive sGenes increased (Supplementary Figure 26b). Furthermore, LC-exclusive sQTLs are associated with intron clusters that have lower read support, when compared to common sQTLs (Supplementary Figure 26c). Hence, this could also indicate an inflated false positive rate associated with LeafCutter quantifications, which has already been observed in differential splicing analyses[13]. For large sample sizes, the number of sQTLs identified by sQTLseekeR2 using LeafCutter or RSEM quantifications is very similar.

We also compared sQTL effect sizes (MD values). In the case of LeafCutter sQTLs, MD values (MD_{LC}) correspond to the absolute maximum difference in mean adjusted intron excision ratios of a given intron cluster (rather than in mean adjusted transcript relative expression, MD_{TQ}, as when using transcript quantifications) between genotype groups. In the case of sGenes with more than one significant intron cluster, sQTL effect sizes were computed as the median MD_{LC} value across all significant intron clusters. As expected, MD_{TQ} values were smaller (median reduction across tissues 7%) for TQ-exclusive sQTLs than for common sQTLs. The same behaviour was observed for the MD_{LC} values of LC-exclusive sQTLs and common sQTLs, although in this case the reduction was much larger (median reduction across tissues 22%). This suggests that LeafCutter quantifications allow to identify smaller effects, and contributes to explain the larger number of LC-exclusive sQTLs with respect to TQ-exclusive sQTLs found. In addition, despite the marked differences in the splicing phenotypes employed, the effect sizes of common sQTLs obtained by LeafCutter (MD_{LC}) and transcript quantifications (MD_{TQ}) displayed a substantial Pearson correlation (median r across tissues of 0.51).

Given that LeafCutter does not provide information about the flanking exons, we could not characterize

the AS events associated with LC-exclusive sQTLs. Nevertheless, we computed the AS events associated with TQ-exclusive and common sQTLs (see section *Alternative splicing events associated with sQTLs* in Methods) and compared them. We observed that the nature of the AS events identified was different between TQ-exclusive and common sQTLs (Supplementary Figure 26d). We further explored individual events using two-sided Wilcoxon Rank-Sum tests to assess the significance of the differences. We found that common sQTLs displayed larger proportions of events involving internal exons, including mutually exclusive exons (*p* value $6.70 \cdot 10^{-10}$) or alternative acceptor (*p* value $4.51 \cdot 10^{-5}$). In contrast, TQ-exclusive sQTLs showed larger proportions of AS events affecting the gene termini, such as alternative first exon (*p* value $1.56 \cdot 10^{-5}$) or alternative 3' UTR (*p* value $4.74 \cdot 10^{-4}$), as well as intron retention (*p* value $7.51 \cdot 10^{-6}$), an event that cannot be identified by LeafCutter. This highlights the complementarity of LeafCutter and transcript quantifications for alternative splicing measurement in the context of sQTL mapping.

Finally, we computed sQTL location (see section *sQTL location* in Methods) for both LC-exclusive and TQ-exclusive sQTLs. Overall, both types of sQTLs displayed a similar distribution along exons, introns and upstream/downstream regions. However, we found a slightly more marked enrichment of TQ-exclusive sQTLs towards the transcription termination site (Supplementary Figure 26e). This may relate to the larger proportion of AS events affecting the gene termini associated with TQ-exclusive sQTLs.

Supplementary Note 3

sQTL mapping in GTEx V8

Transcript expression (TPM) and variant calls (SNPs and short indels) were obtained from the V8 release of the GTEx Project (dbGaP accession phs000424.v8.p2 [https://www.ncbi.nlm.nih.gov/projects/gap/cgi-bin/study.cgi?study_id=phs000424.v7.p2]). These correspond to 15,253 samples from 838 deceased donors with both RNA-seq in up to 54 tissues and WGS data available. In GTEx V8, RNA-seq reads are aligned to the human reference genome (build hg38/GRCh38) using STAR[14] v2.5.3a, based on the GENCODE v26 annotation (https://www.gencodegenes.org/human/release_26.html). Transcript-level quantifications are obtained with RSEM[15] v1.3.0. WGS reads are aligned with BWA-MEM (http://bio-bwa.sourceforge.net) after base quality score recalibration and local realignment at known indels using Picard (http://broadinstitute.github.io/picard). Joint variant calling across all samples is performed using GATK's HaplotypeCaller v3.5. (https://software.broadinstitute.org/gatk/documentation/tooldocs). Further details on GTEx data preprocessing and QC pipelines can be found in 11.

49 tissues with sample size $n \geq 70$ were selected for *cis* sQTL mapping (48 already present in the V7 analysis, plus Kidney Cortex). Gene, transcript and variant filtering was performed as in V7 (see Methods). In total, 4,074,385 variants and 16,202 genes (15,319 protein coding, 883 lincRNA) were analyzed. Analogously, the covariates used were donor ischemic time, gender and age, sample RIN (RNA integrity number), five genotype PCs (see 11 for details), and the WGS platform (Illumina HiSeq 2000 or HiSeq X) plus the library construction protocol (PCR-based or PCR-free) employed. We performed sQTL mapping on each tissue using sqtlseeker2-nf.

At a 0.05 false discovery rate (FDR), we found in GTEx V8 a total of 344,211 *cis* sQTLs affecting 9,051 sGenes (8,662 protein coding genes and 389 lincRNAs). Results are summarized in Supplementary Table 3. As expected, the number of sGenes over the number of tested genes increases with the tissue sample size ($r^2 = 0.88$, Supplementary Figure 27a). In contrast to the V7 analysis, here we observe some signs of saturation for larger sample sizes. The overlap between V8 and V7 sQTLs, evaluated considering the genes tested in both runs, was substantial (median Jaccard index of sGenes across tissues 0.54, Supplementary Figure 27b). Indeed, V7 sQTLs are essentially a subset of V8 sQTLs (median minimum overlap of sGenes across tissues 0.87). The increased sample sizes available in GTEx V8, with respect to GTEx V7, led to the identification of a larger number of sQTLs, with overall smaller effect sizes (Supplementary Figure 27c).

We evaluated the overlap between the V8 sQTLseekeR2 sQTLs and the ones produced by the GTEx Consortium. The GTEx Consortium relies on intron excision ratios obtained by LeafCutter as splicing phenotypes, and on univariate linear regression, as implemented in FastQTL, for association testing[11]. Despite the large differences between the two analyses (regarding the set of variants and genes tested, the filters applied, the length of the *cis* windows selected, the splicing phenotypes and covariates used, the methodology for association testing employed, etc.), we observe a moderate overlap between the two sQTL sets (median

Jaccard index of sGenes across tissues 0.33, median π_1 of sQTLseekeR2's in the Consortium's sQTL-sGene associations across tissues 0.44, Supplementary Figure 27d). Note that the overlap was evaluated considering the variant-gene-tissue trios tested in both runs. Overall, the number of sGenes identified by the GTEx Consortium was larger than the number of sGenes identified by sQTLseekeR2: on average across tissues, out of 100 sGenes, 44 were identified only by the GTEx Consortium, 24 only by us, and 32 in common. The numbers are analogous for sQTLs (i.e. 46, 26 and 28, see also Supplementary Data 7).

A major source for this difference is the nature of the splicing phenotypes employed in the two analyses (RSEM transcript quantifications *vs* LeafCutter intron excision ratios), as we extensively discuss in the Supplementary Note 2. Indeed, the larger number of sQTLs identified by the GTEx Consortium could be explained by the fact that LeafCutter is able to detect novel introns, while our analysis employs RSEM transcript quantifications that rely on GENCODE annotation. It could be also related to an inflated false positive rate associated with LeafCutter quantifications, which has already been described in differential splicing analyses[13]. On the other hand, sQTLseekeR2 added many sQTLs to those identified by the GTEx Consortium (see Supplementary Data 7), likely capturing a population of events that tend to escape detection using the approach based on LeafCutter, as anticipated by our analyses in the Supplementary Note 2. Indeed, when comparing the AS events associated with the sQTLs identified by our approach with those found in common with the GTEx Consortium (note that LeafCutter does not provide information about the flanking exons, and therefore we could not characterize the AS events associated with the sQTLs identified only by the GTEx Consortium), we obtained analogous results (Supplementary Figure 27e) to when comparing RSEM-exclusive *vs* RSEM + LeafCutter sQTLs (Supplementary Figure 26d). In particular, sQTLs identified exclusively by our approach displayed larger proportions of AS events affecting the gene termini (e.g. alternative first exon, two-sided Wilcoxon Rank-Sum test *p* value $2.78 \cdot 10^{-11}$), as well as intron retention (two-sided Wilcoxon Rank-Sum test *p* value $1.47 \cdot 10^{-6}$). The latter is particularly relevant, given that this event, which cannot be identified by LeafCutter, is known to be important in tissues such as blood[16] or brain[17] and has been related to several types of cancer[18]. In contrast, sQTLs identified also by the GTEx Consortium showed larger proportions of events involving internal exons (e.g. exon skipping, two-sided Wilcoxon Rank-Sum test *p* value $< 10^{-16}$).

We further characterized the sQTLs identified only by one of the approaches (either LeafCutter + FastQTL, LCFQ, or RSEM + sQTLseekeR2, RMSQ). For each tissue, we built a dataset with all the approach-exclusive sQTL-sGene associations, and defined the following set of features: number of transcripts of the sGene, number of introns, average number of introns per transcript, average intron length, average exon length, sGene expression (TPM), MAF of the sQTL variant, whether it is present or not in the primary transcript, distance to the TSS, distance to the closest splice site, TFBS, RBP-binding site, GWAS variant, H3K36me3 peak and Pol II binding site. Then, we split the dataset in training and test sets (70/30), and trained a random forest (as implemented in `sklearn.ensemble.randomforestclassifier`) to classify each sQTL-sGene association as LCFQ-exclusive or RMSQ-exclusive. We performed a 10-fold cross-validation with hyperparameter tuning to select the optimal model, which was used to make predictions on the test set. Overall, the classification accuracy was high (average across tissues 98%). We determined the importance of each feature for classifi-

cation as the mean decrease in node impurity from splitting on the feature (measured by the Gini index) and via SHAP values[19] (mean absolute SHAP value). Importance estimates were normalized to sum up to 1. In addition, we employed SHAP values to explore the contribution of each feature to each class at the level of individual observations. In order to evaluate the impact of duplicated feature values in the models, we repeated the analysis after adding gaussian noise to the gene features ($\mathcal{N}(0, \sigma_i/100)$, where σ_i is the standard deviation of gene feature i), leaving the structure of the data unchanged (Supplementary Figure 28a illustrates this for esophagus - gastroesophageal junction, analogous results are obtained for other tissues). The classification accuracy was not affected (average across tissues 98%).

The ranking of the features according to their importance for classification is consistent across tissues (mean Spearman's ρ = 0.97) and importance metrics (mean Spearman's ρ = 0.98), being robust to the addition of gaussian noise to the gene features (mean Spearman's ρ = 0.97). Overall, we identified gene expression, average intron length and average exon length as the most relevant features. The analysis of SHAP values also revealed that lower values of gene expression and average intron length contribute to classify an observation in the RMSQ-exclusive class, while larger values contribute to classification in the LCFQ class. The opposite behaviour is displayed by the average exon length. As regards the variant features, we found that small values of MAF contribute to classify an observation in the RMSQ class (Supplementary Figure 28c-d illustrates these observations for esophagus - gastroesophageal junction, the tissue whose importance estimates are the closest to the mean ones across tissues. Analogous results are obtained for other tissues).

In summary, these results suggest that our approach has larger power to detect sQTLs for genes with lower expression and shorter introns (longer exons), as well as involving variants with smaller MAF. In contrast, the GTEx Consortium's approach would be better powered to detect sQTLs for genes with higher expression and longer introns (shorter exons). A possible explanation for these observations is that both lowly expressed genes and longer introns result in less reliable transcript quantifications and intron-excision ratios, respectively, which could lead to a larger number of associations. Indeed, the former has been reported[20], and as for the latter, we have observed a marked decrease in the number of reads that support an intron as the intron length increases (Supplementary Figure 28e). Regarding MAF, our observation could be due to the fact that in sQTLseekeR2 the genotype is encoded as a categorical variable, while the GTEx Consortium's approach treats it as a numerical variable. Hence, our approach would have larger power to detect what is known in this context as non-additive (either dominant or recessive) genetic effects, especially when there are few individuals in one of the genotype groups.

References

1. Monlong, J., Calvo, M., Ferreira, P. G. & Guigó, R. Identification of genetic variants associated with alternative splicing using sQTLseekeR. *Nature Communications* **5**, 4698 (2014).

2. Anderson, M. A new method for non-parametric multivariate analysis of variance. *Australian Ecology* **26**, 32–46 (2001).

3. Anderson, M. J. & Robinson, J. Generalized discriminant analysis based on distances. *Australian & New Zealand Journal of Statistics* **45**, 301–318 (2003).

4. Davies, R. B. Algorithm AS 155: The Distribution of a Linear Combination of χ^2 Random Variables. *Applied Statistics* **29**, 323 (1980).

5. Montgomery, S. B. *et al.* Transcriptome genetics using second generation sequencing in a Caucasian population. *Nature* **464**, 773–777 (2010).

6. Ongen, H., Buil, A., Brown, A. A., Dermitzakis, E. T. & Delaneau, O. Fast and efficient QTL mapper for thousands of molecular phenotypes. *Bioinformatics* **32**, 1479–1485 (2016).

7. The GTEx Consortium. Genetic effects on gene expression across human tissues. *Nature* **550**, 204–213 (2017).

8. Di Tommaso, P. *et al.* Nextflow enables reproducible computational workflows. *Nature Biotechnology* **35**, 316–319 (2017).

9. Li, Y. I. *et al.* RNA splicing is a primary link between genetic variation and disease. *Science* **352**, 600–604 (2016).

10. Raj, T. *et al.* Integrative transcriptome analyses of the aging brain implicate altered splicing in Alzheimer's disease susceptibility. *Nature Genetics* **50**, 1584–1592 (2018).

11. The GTEx Consortium. The GTEx Consortium atlas of genetic regulatory effects across human tissues. *Science* **369**, 1318–1330 (2020).

12. Li, Y. I. *et al.* Annotation-free quantification of RNA splicing using LeafCutter. *Nature Genetics* **50**, 151–158 (2018).

13. Vaquero-Garcia, J., Norton, S. & Barash, Y. LeafCutter vs. MAJIQ and comparing software in the fast moving field of genomics. *bioRxiv* 463927 (2018).

14. Dobin, A. *et al.* STAR: ultrafast universal RNA-seq aligner. *Bioinformatics* **29**, 15–21 (2013).

15. Li, B. & Dewey, C. N. RSEM: accurate transcript quantification from RNA-Seq data with or without a reference genome. *BMC Bioinformatics* **12**, 323 (2011).

16. Ullrich, S. & Guigó, R. Dynamic changes in intron retention are tightly associated with regulation of splicing factors and proliferative activity during B-cell development. *Nucleic acids research* **48**, 1327–1340 (2020).

17. Mauger, O., Lemoine, F. & Scheiffele, P. Targeted Intron Retention and Excision for Rapid Gene Regulation in Response to Neuronal Activity. *Neuron* **92**, 1266–1278 (2016).

18. Jung, H. *et al.* Intron retention is a widespread mechanism of tumor-suppressor inactivation. *Nature Genetics* **47**, 1242–1248 (2015).

19. Lundberg, S. & Lee, S.-I. A Unified Approach to Interpreting Model Predictions. *Advances in Neural Information Processing Systems* **2017-12**, 4766–4775 (2017).

20. Kanitz, A. *et al.* Comparative assessment of methods for the computational inference of transcript isoform abundance from RNA-seq data. *Genome Biology* **16**, 150 (2015).
