# Supplementary material for: Identification and analysis of splicing quantitative trait loci across multiple tissues in the human genome
Source: Nat Commun. 2021 Feb 1;12:727. doi: 10.1038/s41467-020-20578-2 (PMC7851174; doi:10.1038/s41467-020-20578-2)
Supplement: Supplementary file 2 — Description of Additional Supplementary Files [file 41467_2020_20578_MOESM2_ESM.pdf]

Description of Additional Supplementary Files

File Name: Supplementary Data 1.

Description: Histone mark changes at heteropleiotropic loci. For each heteropleiotropic variant, we report the associated sGene (gene g_1 in tissue t_1), eGene (gene g_2 in tissue t_2), histone mark and tissue where the variant overlaps the mark (either t_1 or t_2).

File Name: Supplementary Data 2.

Description: Values of τ_s and τ corresponding to 469 sGenes under strong tissue-specific splicing regulation ($\tau_s > 0.9325$), 81 of which do not display tissue-specific expression ($\tau < 0.1965$). The τ_s and τ thresholds correspond, respectively, to the top 20 percentile of the distribution of τ_s values and the bottom 20 percentile of the distribution of τ values (see Methods).

File Name: Supplementary Data 3.

Description: Top ten diseases with the highest sQTL enrichment, with respect to non-sQTLs, in the corresponding GWAS loci. We focused on diseases with at least 30 non-sQTLs overlapping their GWAS hits. The odds ratio (OR) for the enrichment is reported. All the enrichments are significant with FDRs $< 2.17 \cdot 10^{-16}$. In each case, we provide references of previous works relating alternative splicing to the disease pathophysiology.

File Name: Supplementary Data 4.

Description: ENTEx ChIP-seq experiments for 6 histone marks across 29 tissues. The accession numbers and URLs provided allow to uniquely identify the experiments on the ENCODE portal (https://www.encodeproject.org).

File Name: Supplementary Data 5.

Description: ENCODE eCLIP experiments for 114 target RBPs in HepG2 and K562 cell lines. The accession numbers and URLs provided allow to uniquely identify the experiments on the ENCODE portal (https://www.encodeproject.org).

File Name: Supplementary Data 6.

Description: ENCODE RNA-seq experiments on nuclear and cytoplasmic fractions of 13 human cell lines. The accession numbers and URLs provided allow to uniquely identify the experiments on the ENCODE portal (https://www.encodeproject.org).

File Name: Supplementary Data 7.

Description: Number of sGenes and sQTLs identified only by our approach (RSEM + sQTLseekeR2, RMSQ), only by the GTEx Consortium approach (LeafCutter + FastQTL, LCFQ), and in common by the two approaches, considering the variant-gene-tissue trios tested in both analyses.
