# Supplementary material for: Identification and analysis of splicing quantitative trait loci across multiple tissues in the human genome
Source: Nat Commun. 2021 Feb 1;12:727. doi: 10.1038/s41467-020-20578-2 (PMC7851174; doi:10.1038/s41467-020-20578-2)
Supplement: Supplementary file 10 — Reporting Summary [file 41467_2020_20578_MOESM10_ESM.pdf]

nature research

Corresponding author(s): Roderic Guigó, Diego Garrido-Martín

Last updated by author(s): Oct 12, 2020

Reporting Summary

Nature Research wishes to improve the reproducibility of the work that we publish. This form provides structure for consistency and transparency in reporting. For further information on Nature Research policies, see Authors & Referees and the Editorial Policy Checklist .

Statistics

For all statistical analyses, confirm that the following items are present in the figure legend, table legend, main text, or Methods section.

n/a	Confirmed	
☐	☒	The exact sample size (*n*) for each experimental group/condition, given as a discrete number and unit of measurement
☐	☒	A statement on whether measurements were taken from distinct samples or whether the same sample was measured repeatedly
☐	☒	The statistical test(s) used AND whether they are one- or two-sided<br>*Only common tests should be described solely by name; describe more complex techniques in the Methods section.*
☐	☒	A description of all covariates tested
☐	☒	A description of any assumptions or corrections, such as tests of normality and adjustment for multiple comparisons
☐	☒	A full description of the statistical parameters including central tendency (e.g. means) or other basic estimates (e.g. regression coefficient) AND variation (e.g. standard deviation) or associated estimates of uncertainty (e.g. confidence intervals)
☐	☒	For null hypothesis testing, the test statistic (e.g. *F*, *t*, *r*) with confidence intervals, effect sizes, degrees of freedom and *P* value noted<br>*Give P values as exact values whenever suitable.*
☒	☐	For Bayesian analysis, information on the choice of priors and Markov chain Monte Carlo settings
☒	☐	For hierarchical and complex designs, identification of the appropriate level for tests and full reporting of outcomes
☐	☒	Estimates of effect sizes (e.g. Cohen's *d*, Pearson's *r*), indicating how they were calculated

Our web collection on statistics for biologists contains articles on many of the points above.

Software and code

Policy information about availability of computer code

Data collection	GTEx data was downloaded from dbGaP (https://www.ncbi.nlm.nih.gov/gap, accessions phs000424.v7.p2, phs000424.v8.p2). ENCODE and ENTEx data was downloaded from the ENCODE portal (www.encodeproject.org). The Ensembl Regulation dataset was downloaded from ftp://ftp.ensembl.org/pub/grch37/release-86/regulation/homo_sapiens/AnnotatedFeatures.gff.gz. The GWAS catalog and the Experimental Factor Ontology (EFO) were downloaded from the European Bioinformatics Institute (EMBL-EBI) website (https://www.ebi.ac.uk). For data download we used IBM Aspera Connect v3.6.1 and GNU Wget v1.14.
Data analysis	The majority of the analyses was performed in R v3.3.2. For sQTL mapping we used the sqtlseeker2-nf pipeline, available at https://github.com/guigolab/sqtlseeker2-nf (DOI 10.5281/zenodo.4065497). A detailed description of the software employed is provided in Methods and the Supplementary Note 1.

For manuscripts utilizing custom algorithms or software that are central to the research but not yet described in published literature, software must be made available to editors/reviewers. We strongly encourage code deposition in a community repository (e.g. GitHub). See the Nature Research guidelines for submitting code & software for further information.

Data

Policy information about availability of data

All manuscripts must include a data availability statement. This statement should provide the following information, where applicable:

- Accession codes, unique identifiers, or web links for publicly available datasets
- A list of figures that have associated raw data
- A description of any restrictions on data availability

All the data employed in this study is publicly available. GTEx data was obtained from dbGaP (https://www.ncbi.nlm.nih.gov/gap), accessions phs000424.v7.p2 (https://www.ncbi.nlm.nih.gov/projects/gap/cgi-bin/study.cgi?study_id=phs000424.v7.p2) and phs000424.v8.p2 (https://www.ncbi.nlm.nih.gov/projects/gap/cgi-bin/study.cgi?study_id=phs000424.v8.p2). ENCODE and ENTEx data was obtained from the ENCODE Portal (www.encodeproject.org, accession numbers and URLs

provided in Supplementary Data 4-6). The Ensembl Regulation dataset was obtained from ftp://ftp.ensembl.org/pub/grch37/release-86/regulation/homo_sapiens/AnnotatedFeatures.gff.gz. The GWAS catalog and the Experimental Factor Ontology (EFO) were obtained from https://www.ebi.ac.uk/gwas and https://www.ebi.ac.uk/efo, respectively. A detailed description of the data can be found in Methods and Supplementary Note 3. The sQTL catalogue generated is available at https://doi.org/10.5281/zenodo.4058759.

Field-specific reporting

Please select the one below that is the best fit for your research. If you are not sure, read the appropriate sections before making your selection.

- [x] Life sciences
- [ ] Behavioural & social sciences
- [ ] Ecological, evolutionary & environmental sciences

For a reference copy of the document with all sections, see nature.com/documents/nr-reporting-summary-flat.pdf

Life sciences study design

All studies must disclose on these points even when the disclosure is negative.

Sample size

Tissue sample size corresponded to the number of samples with both RNA-seq and genotypes available from the GTEx V7 release (dbGaP accession phs000424.v7.p2). Sample sizes were large enough (80-491) for QTL mapping in 48 tissues. The same applies to analyses carried out in GTEx V8 (dbGap accession phs000424.v8.p2), with even larger sample sizes (73-706).

Data exclusions

In GTEx V7, 5 tissues were not analyzed due to their small sample size: kidney cortex (39), bladder (10), fallopian tube (7), ectocervix (6) and endocervix (5). GTEx V8 analyses, however, did include kidney cortex (73).

Replication

To evaluate the replication of the splicing QTLs identified here (in GTEx tissues), we compared them with those obtained in the Blueprint Project for three major human immune cell types. Our sQTLs were highly replicated. Additionally, we used Docker to ensure full reproducibility of our sQTL mapping pipeline. While our sQTL mapping analysis in the GTEx dataset was performed only once, all the attempts at evaluating reproducibility of the containerized pipeline using the test dataset available at https://github.com/guigolab/sqtlseeker2-nf were successful.

Randomization

Randomization was not relevant in this study since there was no group allocation. We controlled for biological and technical confounders by regressing them out from the splicing phenotypes before testing for association with genetic variants.

Blinding

Blinding was not relevant in this study since there was no group allocation.

Reporting for specific materials, systems and methods

We require information from authors about some types of materials, experimental systems and methods used in many studies. Here, indicate whether each material, system or method listed is relevant to your study. If you are not sure if a list item applies to your research, read the appropriate section before selecting a response.

Materials & experimental systems

n/a	Involved in the study	
x		Antibodies
x		Eukaryotic cell lines
x		Palaeontology
x		Animals and other organisms
x		Human research participants
x		Clinical data

Methods

n/a	Involved in the study	
x		ChIP-seq
x		Flow cytometry
x		MRI-based neuroimaging
